# Recapitulating Endochondral Ossification for Bone Repair: From Development to Engineering Strategy

**DOI:** 10.1002/adhm.71155

**Published:** 2026-04-14

**Authors:** Yiqi Su, Zihao He, Qianqian Chen, Long Chen, Caiyi Wei, Zijin Zhou, Jianhao Lin, Hui Li, Dan Xing

**Affiliations:** ^1^ Arthritis Clinic & Research Center Peking University People's Hospital Peking University Beijing China; ^2^ Arthritis Institute Peking University Beijing China; ^3^ Third Clinical College Zhejiang Chinese Medical University Hangzhou China

**Keywords:** bone repair, cartilage, endochondral ossification, engineering strategies, organoid

## Abstract

Endochondral ossification (ECO) is the fundamental mechanism underlying long bone development and fracture healing, driven by a cartilage template that orchestrates highly ordered cellular differentiation, vascular invasion, and matrix remodeling. In recent years, ECO has emerged as a key strategy in bone tissue engineering (BTE) due to its remarkable potential in constructing vascularized bone and repairing large bone defects. This review centers on the strategies to recapitulate ECO, beginning with an overview of the essential biological events and molecular regulatory networks that define this process. We then highlight state‐of‐the‐art strategies for inducing or mimicking ECO in vitro from a tissue engineering perspective, including seed cell selection and programming, biomaterial scaffold design and fabrication, and the delivery and dynamic regulation of bioactive factors, and propose potential improvement strategies based on the limitations of current engineering approaches. In addition, bibliometric analysis is employed to delineate research hotspots and development trends. Looking ahead, the refinement of in vitro engineering strategies may enable precise control of key events in the ECO process, providing more efficient, controllable, and physiologically relevant solutions for large bone defect repair.

## Graphical abstract

This review summarizes the developmental basis of endochondral ossification (ECO) and its applications in bone tissue engineering (BTE). It first outlines the key biological processes and signaling pathways underlying ECO, then discusses biomaterial‐based engineering strategies derived from these principles, and finally highlights future directions for optimizing regenerative engineering approaches.

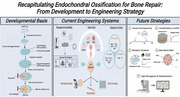

## 1 Introduction

Bone defects are common and challenging clinical problems, particularly in cases of critical‐size bone defects (CSD) or chronic non‐union, where repair outcomes are often suboptimal [1]. Although autografts or allografts can partially fill bone defects, they often fail to reconstruct a complete vascular network and a physiologically relevant microenvironment, and their mechanical strength is usually far from sufficient [2, 3]. In response to these challenges, bone tissue engineering (BTE) strategies and stem cell‐based therapies have emerged [4, 5, 6], becoming prominent research approaches for enhancing bone regeneration. Traditional intramembranous ossification (IMO) strategy primarily relies on the direct differentiation of mesenchymal stem cells (MSCs) into osteoblasts to form bone tissue [7, 8, 9]. However, in complex bone defects, this approach frequently results in insufficient vascularization, incomplete bone regeneration, and poor recovery of mechanical function [10]. Therefore, developing strategies that can effectively enhance vascularization and bone repair efficiency has become an urgent need.

Endochondral ossification (ECO) is a bone formation mechanism distinct from IMO (Table 1) [11, 12, 13, 14, 15, 16, 17]. ECO‐based bone repair strategies closely recapitulate the physiological bone formation observed during embryonic long bone development and fracture healing. Embryonic long bone development follows the classical ECO sequence, including mesenchymal condensation, cartilage template formation and maturation, chondrocyte hypertrophy, vascular invasion, and ossification. During fracture healing, the mode of bone formation is closely influenced by the local mechanical environment. Under conditions of anatomical reduction and rigid fixation, bone repair predominantly proceeds through IMO. However, in most physiologically relevant conditions with relative mechanical stability, IMO and ECO occur concurrently. Specifically, IMO mainly takes place in low‐strain periosteal regions, whereas ECO is favored in mechanically less stable regions such as the fracture gap and external callus, involving the transition from a soft callus to a mineralized hard callus. Consistently, previous studies have demonstrated that low strain and low hydrostatic pressure promote IMO, while moderate strain and hydrostatic pressure favor ECO. In contrast, excessive shear strain often leads to fibrous tissue formation, which is detrimental to bone healing [17, 18, 19]. This mechanosensitive and spatially adaptive nature of bone formation highlights key advantages of ECO in tissue engineering applications. First, the soft callus in ECO serves as a template that supports robust vascular invasion, enabling the formation of highly vascularized bone tissue. Second, the progressive transition from a soft callus to a mineralized hard callus allows ECO to better accommodate dynamic mechanical environments and maintain structural stability during regeneration. Collectively, these features make ECO‐based strategies particularly suitable for BTE, especially for the repair of large and mechanically complex defects.

**TABLE 1:** Comparison of IMO and ECO.

Dimension	IMO	ECO
Developmental Origin	Mesenchymal progenitors	Mesenchymal progenitors
Developmental Pathway	Direct bone formation (without cartilage stage)	Indirect bone formation (via cartilage template)
Vascularization	Accompanied by early‐stage osteogenesis	After cartilage formation, accompanied by hypertrophic maturation
Key Regulatory Factors	RUNX2, OSX, VEGF	SOX9, IHH, VEGF, RUNX2
Mechanobiological regulation in fracture healing	Favored in low‐strain, mechanically stable regions (e.g., periosteum)	Favored in mechanically less stable regions with moderate strain (e.g., fracture gap and external callus)
Major Anatomical Sites	Craniofacial bones, some flat bones	Long bones, cranial base bones, vertebrae
Typical Applications	Craniofacial bone repair	Long bone defect repair

In recent years, significant progress has been made in ECO‐based bone repair research [20, 21, 22, 23, 24, 25, 26]. Current engineering strategies primarily involve three core elements: first, harnessing the potency of stem cells to achieve tissue‐specific differentiation; second, designing functionalized biomaterial scaffolds to provide an appropriate 3D microenvironment that supports cell differentiation, vascularization, and matrix deposition; and third, regulating the local microenvironment with bioactive factors to coordinate osteogenesis, angiogenesis, and tissue remodeling [23, 25, 27, 28, 29, 30, 31, 32, 33, 34, 35]. Based on these three elements, researchers have developed “ECO organoids” through engineering approaches, which are in vitro constructs capable of recapitulating key events of ECO and providing controllable experimental models for repairing large bone defects. However, several challenges remain. For example, although in vitro ECO organoids can partially reproduce the bone formation microenvironment, limitations persist in vascularization, mechanical microenvironment regulation, and the stability of spatiotemporal signaling gradients. In addition, the design of functionalized scaffolds and bioactive factor delivery systems still struggles to achieve precise, coordinated control over cell differentiation, vascularization, and bone matrix deposition. Furthermore, the selection, expansion, and directed differentiation of seed cells, as well as the interaction mechanisms among multiple cell lineages (chondrocytes, osteoblasts, endothelial cells, et al.) [36], remain incompletely understood. Finally, challenges persist in terms of the controllability, scalability, and clinical translation of engineered bone constructs. Thus, a systematic review of existing ECO engineering strategies is essential to inform the design of efficient, controllable, and clinically relevant BTE solutions.

Although ECO‐based engineering strategies have become increasingly mature, they still face numerous challenges, highlighting the need for a deeper understanding of the underlying biological mechanisms of ECO. To address these gaps, we systematically summarize the biological events and signaling regulations of ECO, comprehensively review recent engineering strategies to recapitulate it, and propose potential optimization approaches. In addition, bibliometric analysis is employed to delineate current research hotspots and emerging trends. This perspective not only helps elucidate the mechanisms of bone repair but also provides theoretical and practical guidance for the refinement of engineering strategies and their clinical translation.

## 2 Biological Processes of ECO

**FIGURE 1:**
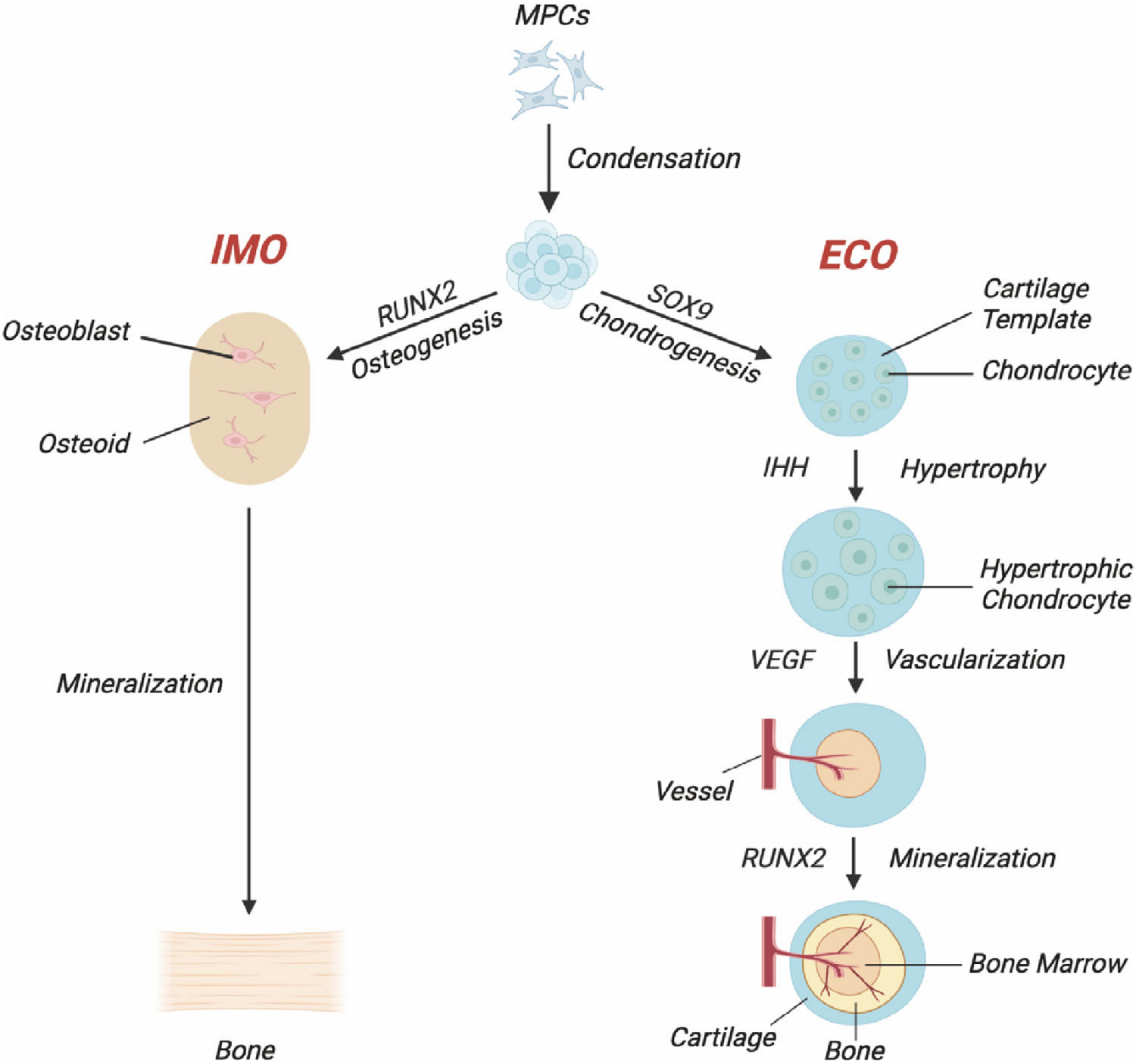
Biological processes of IMO and ECO during embryonic development. Mesenchymal condensation is the first step that initiates skeletal development. In IMO, MPCs differentiate directly into osteoblasts, which deposit osteoid that subsequently mineralizes into mature bone. ECO begins with the condensation of MPCs and their differentiation into chondrocytes, forming a cartilage template. As chondrocytes mature, they enter the hypertrophic stage, and the internal region develops a hypoxic, low‐cell‐density microenvironment that signals subsequent vascular invasion and ossification. Hypertrophic chondrocytes secrete angiogenic factors and promote vascular invasion. The invading blood vessels deliver osteoprogenitor cells and osteoclast precursors, participating in matrix mineralization and remodeling. Gradually, the calcified cartilage region is replaced by trabecular bone, ultimately establishing a mature bone marrow cavity. The figure was created using BioRender.com.

ECO primarily occurs during the development of the appendicular and axial skeleton, including long bones, cranial base bones, and vertebrae [37, 38]. This process is a highly ordered, multistep developmental program that begins with the differentiation of mesenchymal progenitor cells (MPCs), which can give rise to multiple lineages, including chondrocytes, osteoblasts, and adipocytes [39]. It should be noted that here MPCs referred to here are embryonic or developmental‐stage progenitor cells, which differ from the adult MSCs commonly used in tissue engineering in terms of origin, differentiation potential, and physiological state, although the definition of MSCs remains controversial [40, 41]. Unlike the direct osteogenic differentiation seen in IMO, During ECO, MPCs first condensation and differentiate to form a cartilage template, followed by chondrocyte hypertrophy, vascular invasion, and matrix mineralization, ultimately culminating in bone formation, with each stage finely regulated by complex signals (Figure 1) [42]. A thorough understanding of the key biological events in ECO not only helps elucidate the intrinsic mechanisms of skeletal development but also provides a theoretical foundation for the design of in vitro engineering constructs and in vivo bone tissue repair strategy, thereby guiding controllable, engineering‐based strategies for regenerative medicine.

### 2.1 Condensation

During embryonic development, progenitors first migrate to the prospective skeletal sites. Following sufficient proliferation, they undergo a critical condensation phase, marking their earliest lineage commitment and providing a prerequisite for chondrogenic differentiation. This process involves cytoskeletal reorganization, extracellular matrix (ECM) remodeling, and enhanced cell–cell adhesion [43, 44, 45]. Significant understanding of the signaling pathways involved in mesenchymal condensation was established decades ago. Among the early inductive cues, transforming growth factor‐β (TGF‐β) upregulates the expression of neural cadherin (N‐cadherin) and neural cell adhesion molecule (N‐CAM), thereby promoting close intercellular contacts, accompanied by increased expression of peanut agglutinin (PNA)‐binding surface markers. Moreover, the condensation process is also regulated by bone morphogenetic protein (BMP) and fibroblast growth factor (FGF) signaling and involves genes such as the HOX gene family, PAX‐1/9, and PRX‐1/2, along with numerous other transcription factors that collectively govern cell adhesion, proliferation, and differentiation. Proteins including syndecan, tenascin, and noggin play key roles in determining the termination of condensation and establishing the boundaries of the mesenchymal condensates. As a result of condensation, the mesenchymal cell density in the limb bud core increases substantially and creates the required hypoxic environment, which subsequently activates essential chondrogenic genes such as sex‐determining region Y (SRY)‐box transcription factor 9 (SOX9), thereby initiating overt chondrogenic differentiation [43, 46, 47, 48, 49, 50]. Notably, the timely downregulation of N‐cadherin following condensation is crucial for the onset of chondrocyte differentiation [51]. Conversely, failure of condensation disrupts the microenvironment and impairs chondrogenesis. Animal studies have shown that conditional deletion of CDH2 (encoding N‐cadherin) leads to reduced bone mass and decreased numbers of osteoprogenitor cells [52].

### 2.2 Chondrogenesis

After condensation, MSCs begin to differentiate into chondrocytes under the precise regulation of multiple signaling pathways, with the activation of the transcription factor SOX9 playing a pivotal initiating role. The newly differentiated chondrocytes secrete an ECM rich in type II collagen (COL‐II) and aggrecan (ACAN), gradually constructing a cartilage template (also called cartilage primordium). This transient template not only provides structural support for early skeletal morphology but also functions as a dynamic signaling hub, integrating genetic programs and biomechanical cues to regulate tissue development, while progressively exhibiting pronounced cellular heterogeneity as differentiation proceeds. During the subsequent stages of chondrocyte hypertrophy and ossification, the cartilage template coordinates the spatial organization and differentiation of tissues, laying the foundation for bone formation [23, 44, 53, 54, 55, 56].

### 2.3 Chondrocyte Hypertrophy

Within the cartilage template, chondrocytes undergo gradual differentiation and maturation, ultimately entering the hypertrophic stage. This process is considered the principal mechanism driving longitudinal bone growth during long bone development [57, 58], with the initial molecular events marked by downregulation of SOX9 and upregulation of Indian Hedgehog (IHH) and runt‐related transcription factor 2 (RUNX2), involving coordinated changes in both cells and ECM [14, 59]. During this stage, chondrocytes markedly increase in volume—by approximately 10–20 fold—forming hypertrophic chondrocytes (HyCs), accompanied by extensive metabolic and transcriptional reprogramming. Studies have shown that chondrocyte volume expansion occurs in three consecutive phases: the first phase involves proportional accumulation of dry mass and fluid; the second phase is characterized by rapid cell swelling due to water uptake; and the third phase involves coordinated volumetric growth of both dry mass and fluid, resulting in stable expansion. The longitudinal growth rate of different growth plates largely depends on the duration of this third phase [60]. Meanwhile, the ECM composition gradually shifts from being predominantly COL‐II to type X collagen (COL‐X), transitioning from a hyaline cartilage matrix to a hypertrophic cartilage matrix [61]. As the avascular cartilage template thickens, its central region gradually experiences hypoxia and nutrient limitation, resulting in an area of low cell density. At the same time, this hypoxic microenvironment provides signals that promote subsequent vascular invasion and ossification. The hypertrophic zone of cartilage exhibits a complex paracrine signaling and ECM remodeling microenvironment, providing the necessary structural and signaling foundation for subsequent vascular invasion and matrix mineralization [23, 62, 63, 64, 65].

### 2.4 Vascular Invasion

Angiogenesis represents a critical bridging stage during endochondral bone formation. In addition to COL‐X, HyCs secrete matrix‐degrading enzymes such as matrix metalloproteinase 13 (MMP13), which promote the breakdown of the pre‐existing hyaline cartilage matrix and thereby create the physical space required for subsequent vascular invasion into the hypertrophic zone [59, 66, 67]. Simultaneously, matrix degradation is accompanied by an upregulation of pro‐angiogenic signals. Under stimuli such as local hypoxia, HyCs markedly increase the production and secretion of pro‐angiogenic factors, most notably hypoxia‐inducible factor 1‐alpha (HIF‐1α) and vascular endothelial growth factor (VEGF), with the latter serving as the key signal that drives endothelial cells (ECs) migration and neovascularization. Driven by this shift in the local microenvironment, newly formed blood vessels grow into the hypertrophic region, initiating the preliminary vascularization. Successful vascular invasion marks the establishment of the primary ossification center (POC), which essentially corresponds to the formation of the primitive marrow cavity [36, 68]. As blood vessels penetrate the hypertrophic zone, oxygen, nutrients, and various growth factors are delivered to the site, accompanied by the recruitment of multiple cell populations, including osteoprogenitors and osteoclast precursors. This transition renders the local niche increasingly complex, where diverse cell types interact and form intricate metabolic coupling [69, 70, 71, 72]. Signals regulating osteogenesis, angiogenesis, and osteoclastogenesis collectively establish a dynamically remodeling equilibrium that drives the conversion of cartilage into mature bone tissue.

Previously, due to limitations in bone tissue processing techniques, the vascular morphology within bone was difficult to fully characterize. Recent studies using advanced methods have successfully identified a specialized subtype of capillaries with distinct structural and molecular features: type H vessels (CD31+EMCN+). These vessels are predominantly located at the metaphysis, where RUNX2+ and Osterix+ progenitors are largely distributed, indicating a tight coupling with bone metabolic activity and forming a coordinated angiogenesis–osteogenesis network. In contrast, type L vessels are primarily found within the bone marrow cavity, exhibiting lower metabolic activity but playing a critical role in supporting hematopoiesis. Type H vessels dramatically decrease with age and are gradually converted into quiescent type L vessels, thereby limiting bone growth activity in the elderly [73, 74]. Collectively, type H vessels play a key role in growth plate metabolism, facilitating the conversion of cartilage to bone through their coupling with osteogenesis.

### 2.5 Osteogenesis and Matrix Mineralization

During long bone development, the earliest osteogenic events do not occur within the hypertrophic cartilage region but originate in the perichondrium located at the mid‐portion of the cartilaginous anlage. This region acquires vascular supply earliest, and under specific signaling cues, osteoprogenitor cells in perichondrium initiate IMO, forming a circumferential bone collar that gradually extends bilaterally [75]. As blood vessels invade the internal hypertrophic cartilage region, additional osteoprogenitors are delivered to the site. Under the regulation of transcription factors such as RUNX2, they differentiate into osteoblasts and express osteogenic markers including alkaline phosphatase (ALP), type I collagen (COL‐I), osteocalcin (OCN), and osteopontin (OPN). Osteoblasts deposit mineralized matrix, progressively replacing the cartilage template and forming primitive trabeculae (woven bone) [14, 68].

Recent lineage‐tracing studies have shown that a subset of HyCs do not undergo apoptosis; instead, under specific signaling regulation, they can directly transdifferentiate into osteoblasts and persist into adulthood [76, 77, 78]. This finding highlights that osteoblasts generated during the late stages of ECO are not exclusively derived from osteoprogenitor cells recruited via invading blood vessels. In both embryonic development and fracture healing, late‐stage ECO involves a dual source of osteoblasts: (1) differentiation of osteoprogenitor cells delivered via invading blood vessels, and (2) direct transdifferentiation of a subset of hypertrophic chondrocytes. In recent years, this dual‐origin concept has been further supported and elaborated by numerous studies [11, 78, 79], highlighting its importance for understanding bone formation and for developing regenerative strategies.

Concurrent with osteogenesis is osteoclast‐mediated matrix remodeling. Invading blood vessels bring osteoclast precursors, which differentiate into osteoclasts and resorb calcified cartilage, creating space for the ordered deposition of bone matrix and contributing to subsequent remodeling of primitive trabeculae. Meanwhile, osteoclasts originating from the endosteum continue to resorb internal bone tissue, expanding the marrow cavity and shaping the developing bone. Through the gradual establishment of a dynamic balance between osteoblast and osteoclast activity, bone deposition and resorption become spatially and temporally coordinated, allowing newly formed woven bone to be remodeled into mature bone with stable mechanical properties [80, 81].

Insights gained from developmental ECO provide critical guidance for bone repair and tissue engineering. Key processes such as chondrogenic differentiation, chondrocyte transdifferentiation, vascular invasion, and matrix remodeling not only inspire engineering strategies but also define specific requirements. First, efficient mesenchymal condensation and chondrogenesis form the foundational basis for successful engineering constructs. Second, the differentiation trajectory from MSCs to chondrocytes and subsequently to osteoblasts must be tightly and precisely regulated. Third, vascularization during later stages is crucial and may require the rational incorporation of pro‐angiogenic factors and/or endothelial (progenitor) cells. Fourth, the physicochemical properties of scaffolds must be carefully tuned to ensure orderly matrix remodeling. By understanding and integrating these principles, tissue engineering approaches can aim to recapitulate the ECO sequence both in vitro and in vivo, promoting the formation of bone tissue that closely mimics native structure and function and faithfully reproduces physiological development.

## 3 Transcription Factors and Signaling Pathways in ECO

Transcription factors and signaling pathways are the core mechanisms through which cells sense environmental cues and regulate gene expression, precisely guiding each stage of ECO. SOX9 and RUNX2 play key roles in chondrogenesis and osteogenic differentiation, respectively, while TGF‐β, Hedgehog, VEGF, FGF, Notch, WNT, and mechanotransduction pathways interact synergistically or antagonistically to form a complex regulatory network that directs mesenchymal cells toward cartilage and bone lineages [14, 59, 82]. A deeper understanding of this network is crucial for elucidating the molecular mechanisms of ECO and for designing effective BTE strategies.

### 3.1 SOX9

In the early stage of ECO, SOX9 drives mesenchymal condensation and chondrogenic differentiation by activating cartilage‐specific genes such as COL2A1 and ACAN [53]. A recent study has shown that SOX9+ sclerotomal progenitors possess robust chondrogenic regenerative potential [55]. During the proliferative phase, its sustained expression maintains the chondrocytes’ phenotype and suppresses premature hypertrophy by inhibiting RUNX2. As ECO progresses, SOX9 expression decreases in chondrocytes, relieving repression of hypertrophic markers such as COL10A1 and promoting hypertrophy [83, 84]. Interestingly, although SOX9 mRNA is rapidly downregulated at this stage, residual SOX9 protein persists and remains essential for hypertrophy, as its loss leads to failure of chondrocyte hypertrophy and COL10A1 expression [85]. Overall, the functional roles of SOX9 in ECO are well established. In recent years, research has increasingly focused on the regulatory mechanisms governing its expression, including epigenetic regulation, non‐coding RNA‐mediated control, and regulation of protein stability [86, 87, 88, 89]. These findings indicate that SOX9 is precisely controlled by a multilayered regulatory system and occupies a central position in the network governing chondrocyte fate. In tissue engineering, SOX9 is commonly used to maintain the chondrocyte phenotype and promote cartilage regeneration [90, 91].

### 3.2 RUNX2

RUNX2 is a key transcription factor in bone formation and regulates chondrocyte hypertrophy and osteogenic differentiation during ECO. During the osteogenic phase, RUNX2 further drives osteoblast differentiation and activates downstream target genes, including the transcription factor Osterix (also called OSX or Sp7) and bone matrix proteins COL1A1, OCN, OPN, and BSP (bone sialoprotein), facilitating bone matrix production [92, 93]. However, RUNX2 exerts a negative effect on osteoblast maturation, as its expression is downregulated in late‐stage osteoblasts [94]. It should be noted that RUNX2 is also essential for chondrocyte proliferation. Cartilage lacking RUNX2 does not form proliferative or hypertrophic zones [95]. These findings highlight the critical importance of RUNX2 during ECO. Although the fundamental functions of RUNX2 in ECO have been well elucidated, recent studies have focused on the fine‐tuned regulation of its activity. Key mechanisms include alternative splicing to maintain the balance between functional and non‐functional RUNX2 isoforms [96]; post‐translational regulation and cofactor interactions fine‐tune RUNX2 activity [97, 98]; nuclear transport to achieve proper subcellular localization [99]; and modulation of chromatin accessibility at RUNX2 target genes [100]. Overall, these findings indicate that RUNX2 is precisely controlled by a multilayered regulatory network, ensuring proper osteoblast differentiation during the late stage of ECO. In regenerative strategies, modulating RUNX2 activity can enhance regeneration outcomes [101, 102].

### 3.3 TGF‐β Signaling Pathway

The BMP/TGF‐β signaling pathways are key regulators of ECO. BMP signaling exerts positive effects at all stages of ECO, whereas the role of TGF‐β signaling is stage‐dependent: during the early phase of ECO, TGF‐β enhances the expression and activity of SOX9 through ALK5‐SMAD2/3 activation, promoting cartilage matrix deposition; in addition, it restricts the differentiation of the osteoblast lineage, maintaining the homeostasis of cartilage progenitors. In the later phase of differentiation, TGF‐β signaling promotes terminal chondrocyte differentiation and matrix mineralization via ALK1‐SMAD1/5/8 [103, 104, 105]. TGF‐β/SMAD signaling is further modulated by the inhibitory factor SMAD7, which limits excessive signal activation [106]. Overall, it regulates ECO in a stage‐dependent manner, maintaining cartilage formation and proliferation during the early phase while modulating cartilage‐to‐bone transition in the later phase. The BMP–IHH–FGF regulatory loop promotes chondrocyte proliferation, restrains premature hypertrophy, and modulates RUNX2 activity, thereby ensuring the temporal and spatial coordination of ECO. In contrast, TGF‑β inhibits IHH expression while promoting PTHrP expression, thereby enhancing chondrocyte proliferation and suppressing hypertrophic differentiation [103, 107]. The BMP/TGF‑β signaling pathways have been widely applied to regulate MSC differentiation and in cartilage/bone tissue engineering studies, often applied for in vitro differentiation induction or as delivery systems to enhance tissue regeneration [103, 108, 109, 110].

### 3.4 Hedgehog Signaling Pathway

Hedgehog signaling comprises three ligands (SHH, DHH, and IHH), among which IHH plays a central role in skeletal development. IHH is primarily secreted by prehypertrophic chondrocytes, and its signal transduction involves binding to the receptor Patched (PTCH), relieving inhibition of Smoothened (SMO), and ultimately activating GLI to regulate downstream gene expression [111, 112]. Studies have shown that loss of IHH will suppress chondrocyte proliferation and differentiation. In contrast, constitutive activation of IHH results in excessive bone growth [113]. During endochondral bone development, IHH forms a classic IHH–PTHrP negative feedback loop with parathyroid hormone‐related protein (PTHrP): IHH promotes PTHrP expression, while PTHrP in turn inhibits premature entry of chondrocytes into hypertrophy, thereby maintaining the balance between proliferative and hypertrophic zones [14, 112, 114]. Consequently, the IHH–PTHrP loop guides chondrocyte hypertrophy at the appropriate timing, ensuring the stability and rhythmicity of longitudinal bone growth. Recent studies have further revealed multilayered regulation of Hedgehog signaling, encompassing chondrocyte proliferation, hypertrophy, and osteogenic differentiation [115, 116, 117, 118, 119]. Moreover, non‐canonical, SMO‐independent regulatory mechanisms of Hedgehog signaling have been recently identified [113]. These findings highlight IHH as a pivotal signal in the cartilage‐to‐bone transition during ECO and suggest potential intervention points for regenerative strategies to optimize cartilage and bone repair.

### 3.5 VEGF Signaling Pathway

Angiogenesis is a critical step linking chondrocyte hypertrophy to bone formation and a key factor for robust bone formation. Among the multiple signaling pathways involved, VEGF plays a particularly critical role, along with HIF‐1α, SLIT3 (slit guidance ligand 3), PDGF (platelet‐derived growth factor), Notch, FGF, and others. HyCs express HIF‐1α under hypoxic conditions, which in turn markedly upregulates the secretion of VEGF, acting on ECs through vascular endothelial growth factor receptor 2 (VEGFR2), and promoting vascular invasion into the hypertrophic cartilage region and subsequent matrix remodeling. This process represents a core mechanism of vascularization during endochondral bone development. Besides that, SLIT3, PDGF, Notch, and FGF signals can also regulate angiogenesis and osteogenesis, coordinating vascular growth and bone formation [36, 120, 121, 122].

**FIGURE 2:**
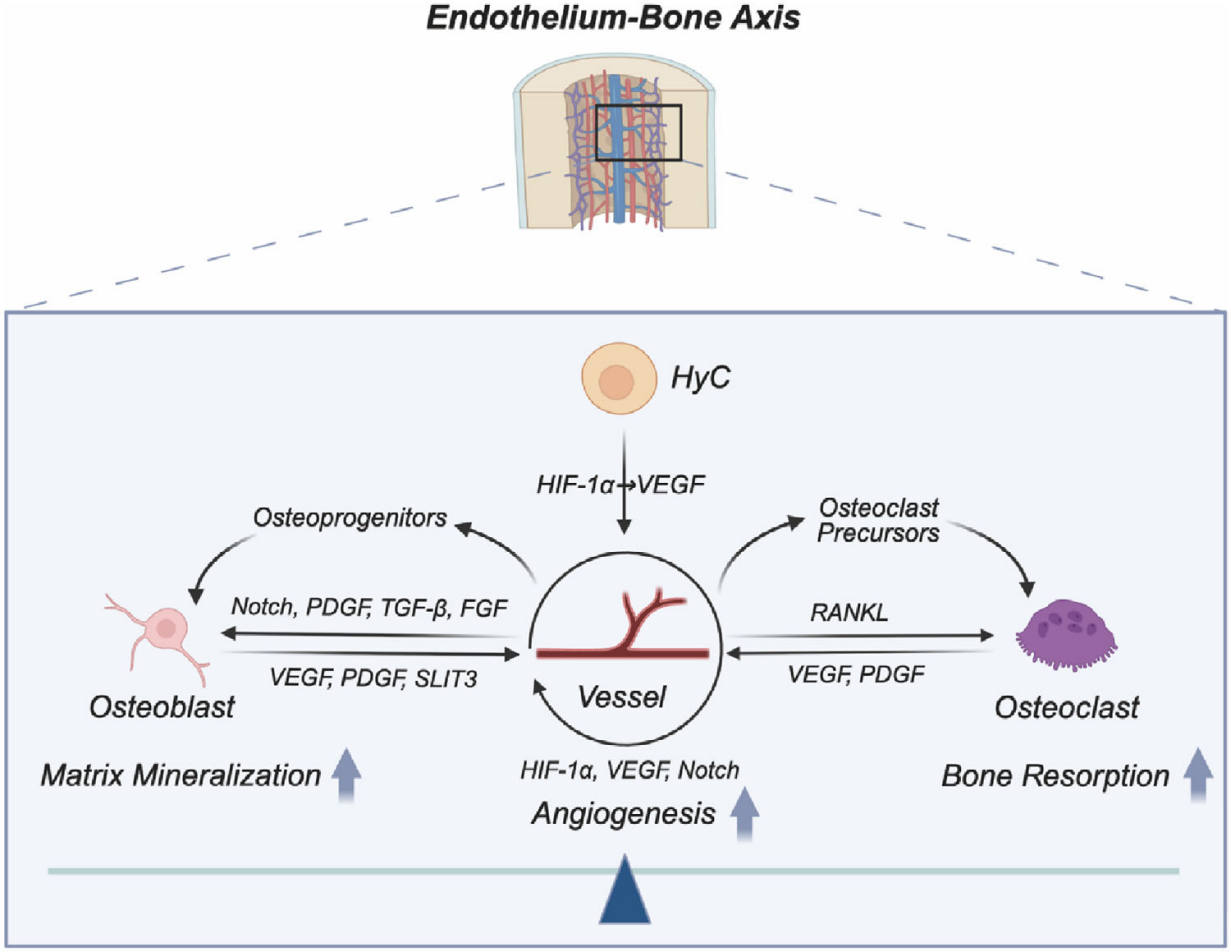
Vasculature‐centered endothelial‐bone axis. In the late stage of ECO, HyCs, osteoblasts, and osteoclasts contribute to angiogenesis via VEGF secretion, while osteoblasts and osteoclasts additionally produce PDGF, and osteoblasts further secrete SLIT3 to enhance vascular formation. Conversely, type H‐ECs promote osteogenic activity via Notch, PDGF, TGF‐β, and FGF signals. Moreover, EC‐derived RANKL induces osteoclast differentiation, coordinates bone resorption, and facilitates dynamic remodeling of cartilage–bone tissue. The figure was created using BioRender.com.

Type H vessels are present in the growth plate and provide strong support for osteogenesis, maintaining coordinated development of the vasculature and bone tissue; this process is referred to as osteogenesis‐angiogenesis coupling. HyCs, osteoblasts, and ECs secrete VEGF, osteoclast precursors secrete PDGF‐BB, and mature osteoblasts and osteoclasts secrete SLIT3; together, these factors synergistically promote the formation of type H vessels. PDGF‐BB also facilitates the migration, proliferation, and differentiation of MSCs and endothelial progenitor cells. The spatiotemporal dynamics of VEGF and PDGF‐BB signaling are crucial for vascular maturation [36, 120, 123, 124, 125]. Moreover, some FGF family members may promote osteogenesis and vascularization by activating VEGF signaling [126]. Type H‐ECs highly express PDGF, TGF‐β, and FGF, providing proliferative and survival signals to osteoprogenitors and further enhancing osteogenesis [73]. They also secrete MMPs to promote ECM degradation in the hypertrophic cartilage zone, and express RANKL, which regulates vessels‐associated osteoclasts (VAOs) through RANKL‐RANK signaling, promoting cartilage resorption and bone formation while supporting the anastomosis of vessels [72]. Notch and its ligand Dll4 transmit signals via cell–cell contact between ECs, a process regulated by blood flow; overactivation of Notch signaling in bone ECs enhances angiogenesis and osteogenesis, whereas reduced blood flow diminishes Notch signaling, leading to decreased bone mass and impaired vascularization [121, 127]. Overall, this complex signaling crosstalk network collectively establish the endothelial‐bone axis, ensuring the formation of vascularized bone tissue during the later stages of ECO (Figure 2). Therefore, a comprehensive understanding of the vascularization mechanisms and the osteogenesis‐angiogenesis coupling is essential for the engineering of endochondral bone tissue.

### 3.6 FGF Signaling Pathway

FGF family signaling through FGFRs exerts complex, stage‐ and location‐specific effects during skeletal development. FGFs involved in cartilage metabolism include FGF1, FGF2, FGF8, FGF9, FGF18, and FGF23, among which the FGF18–FGFR3 axis is the most extensively studied in the context of endochondral bone development [122]. FGF18 is a classic ligand of FGFR3, and current studies suggest that its functions differ between the growth plate and articular cartilage. In the growth plate, FGF18 exhibits temporal specificity: it promotes chondrocyte proliferation and differentiation during early development but switches to an inhibitory role at later stages. In addition to binding FGFR3, FGF18 can also suppress the IHH–PTHrP pathway, thereby negatively regulating chondrocyte proliferation and differentiation. By contrast, in articular cartilage, FGF18 markedly promotes cartilage proliferation and inhibits matrix degradation, at least in part by suppressing Noggin and thereby enhancing BMP‐mediated chondrogenic activity [128, 129, 130, 131, 132, 133]. Recombinant human FGF18 (rhFGF18, Sprifermin) has been validated as a promoter of cartilage regeneration in osteoarthritis (OA) [134]. Recent studies have shown that FGF signaling also participates in cartilage and bone development by regulating autophagy and programmed cell death [135, 136, 137, 138]. In regenerative applications, FGF signaling has been widely utilized to enhance tissue repair, particularly FGF2 and FGF18 [132, 139, 140]. However, given the complex spatiotemporal functions of this signaling pathway, its application in tissue engineering still requires further validation.

### 3.7 WNT Signaling Pathway

The WNT signaling pathway is a key regulator of skeletal development and remodeling, encompassing the canonical WNT/β‑catenin pathway as well as noncanonical pathways. The role of WNT signaling during ECO is now relatively well established, primarily promoting chondrocyte hypertrophy and osteogenesis through synergistic regulation of BMP, IHH, RUNX2, and OSX signaling. Impaired WNT function negatively affects bone mass [141, 142, 143, 144]. Moreover, WNT signaling participates in osteocyte mechanotransduction and the regulation of osteoclast differentiation [145, 146, 147]. However, it has been reported that excessive WNT activation can inhibit early cartilage formation and disrupt growth plate structure and function [148, 149]. A recent study has shown that inhibition of canonical WNT ligands can promote cartilage differentiation [150]. Clinically, monoclonal antibodies targeting sclerostin (Romosozumab), a natural WNT inhibitor, have been used to treat osteoporosis [151]. An increasing number of studies have shown that modulation of WNT signaling holds potential for enhancing bone regeneration in tissue engineering contexts [152, 153, 154].

### 3.8 RhoA/ROCK Signaling Pathway

Osteogenic lineage cells are the classical mechanosensitive cells. In addition, chondrocytes and osteoclast (precursor) cells have also been experimentally shown to possess the ability to sense mechanical signals [155, 156]. RhoA/ROCK‐mediated mechanotransduction is increasingly implicated in skeletal development and regeneration. For example, mechanical tension promotes bone formation through the RhoA/ROCK‐TAZ axis, whereas loss of RhoA impairs osteogenesis under mechanical loading [157]. Additionally, activation of the RhoA/ROCK pathway enhances osteogenic differentiation of MSCs [158]. RhoA also regulates bone remodeling by promoting osteoclastogenesis [159]. Moreover, aberrant activation of RhoA/ROCK negatively affects cartilage by inducing ECM degradation and chondrocyte dysfunction, highlighting its role in cartilage degeneration and its potential as a therapeutic target [160]. Understanding RhoA/ROCK‐mediated mechanotransduction provides a theoretical basis for tissue engineering strategies, particularly in designing biomaterials and mechanical stimulation regimes to optimize bone and cartilage regeneration. Other mechanosensitive signals, such as PIEZO1, have been increasingly shown to be closely associated with bone and cartilage metabolism in recent studies [161, 162, 163, 164].

**FIGURE 3:**
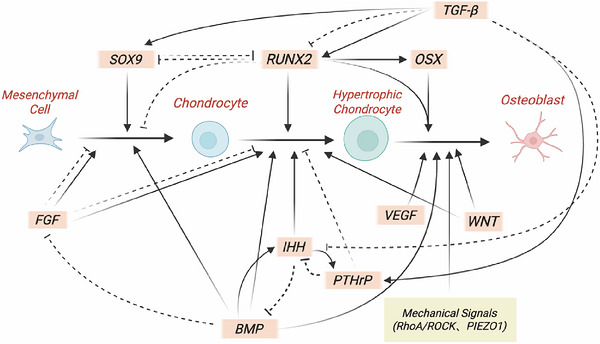
Key transcription factors and signaling pathways during ECO and their general relationships. This figure illustrates the core differentiation axis in ECO, from mesenchymal cells to chondrocytes, hypertrophic chondrocytes (HyCs), and ultimately osteoblasts. SOX9 and RUNX2 serve as the central transcription factors, regulating chondrogenesis and chondrocyte hypertrophy/osteogenic transdifferentiation, respectively. Key signaling molecules, including BMP, TGF‑β, IHH, PTHrP, FGF, WNT, and VEGF, as well as mechanical signals represented by RhoA/ROCK and PIEZO1, interact through synergistic or antagonistic mechanisms to finely control cell fate decisions and matrix remodeling at each stage, ensuring orderly transition from cartilage to bone. The figure was created using BioRender.com.

Mechanical and biochemical signals are intricately intertwined, jointly regulating cell fate and matrix remodeling. Mechanical stimuli activate downstream signaling and act in concert with biochemical cues such as BMP, TGF‑β, IHH, PTHrP, FGF, WNT, and VEGF to precisely control the temporal progression of ECO (Figure 3). At the same time, interactions between cells and biomaterials (e.g., scaffolds or ECM mimics) as well as the growth factors presented on these substrates can significantly influence mechanical and biochemical signal transduction, thereby modulating cell differentiation and tissue regeneration efficiency. A deeper understanding of the molecular regulatory network of ECO can provide valuable guidance for coupling seed cells, biomaterial scaffolds, and bioactive factors in BTE strategies (Table 2).

**TABLE 2:** Key regulators of ECO and their tissue engineering applications.

Factor/Pathway	Main function in ECO	Tissue engineering application
SOX9	Promoting chondrogenesis and maintaining chondrocyte phenotype	Constructing cartilage templates
RUNX2	Driving chondrocyte hypertrophy and osteogenic differentiation	Enhancing bone formation
BMP/TGF‐β	Promoting chondrogenesis and osteogenesis	Delivering via growth factors or scaffolds to promote cartilage/bone regeneration
Hedgehog	Regulating chondrocyte hypertrophy and cartilage‐to‐bone transition	Targeting to control timing of hypertrophy and osteogenesis in engineered tissues
VEGF	Promoting angiogenesis coupled with osteogenesis	Delivering via scaffolds or gene therapy to enhance vascularized bone constructs
FGF	Regulating chondrocyte proliferation, differentiation, and osteogenesis	Delivering to enhance cartilage regeneration
WNT	Promoting chondrocyte hypertrophy, osteogenesis, and mechanotransduction	Modulating with small molecules or biomaterials to improve bone formation
RhoA/ROCK	Mediating mechanotransduction, promoting osteogenesis	Designing biomaterial scaffolds and applying mechanical forces

## 4 Existing Tissue Engineering Strategies for ECO

In recent years, BTE strategies have increasingly focused on mimicking and reconstructing the natural ECO process to achieve more biomimetic and effective bone regeneration. Based on the understanding outlined above of ECO's temporal events and signal regulation, various engineering strategies have been developed, primarily centered on three core components: seed cells, biomaterial scaffolds, and bioactive factors. Seed cells provide the differentiation potential and cellular source required for engineering constructs. Scaffolds serve as 3D structural and microenvironmental frameworks that support cell adhesion, proliferation, and differentiation, while further modulating cell fate transitions through physical, chemical, or mechanical cues. Bioactive factors act as exogenously applied signaling molecules that activate specific pathways to induce or enhance particular biological processes. The appropriate integration of these three elements constitutes a comprehensive engineering system that drives the formation of cartilage, chondrocyte hypertrophy, vascularization, and ultimately ossification, providing a systematic framework to recapitulate the natural ECO process and guide bone regeneration.

### 4.1 Seed Cells

**FIGURE 4:**
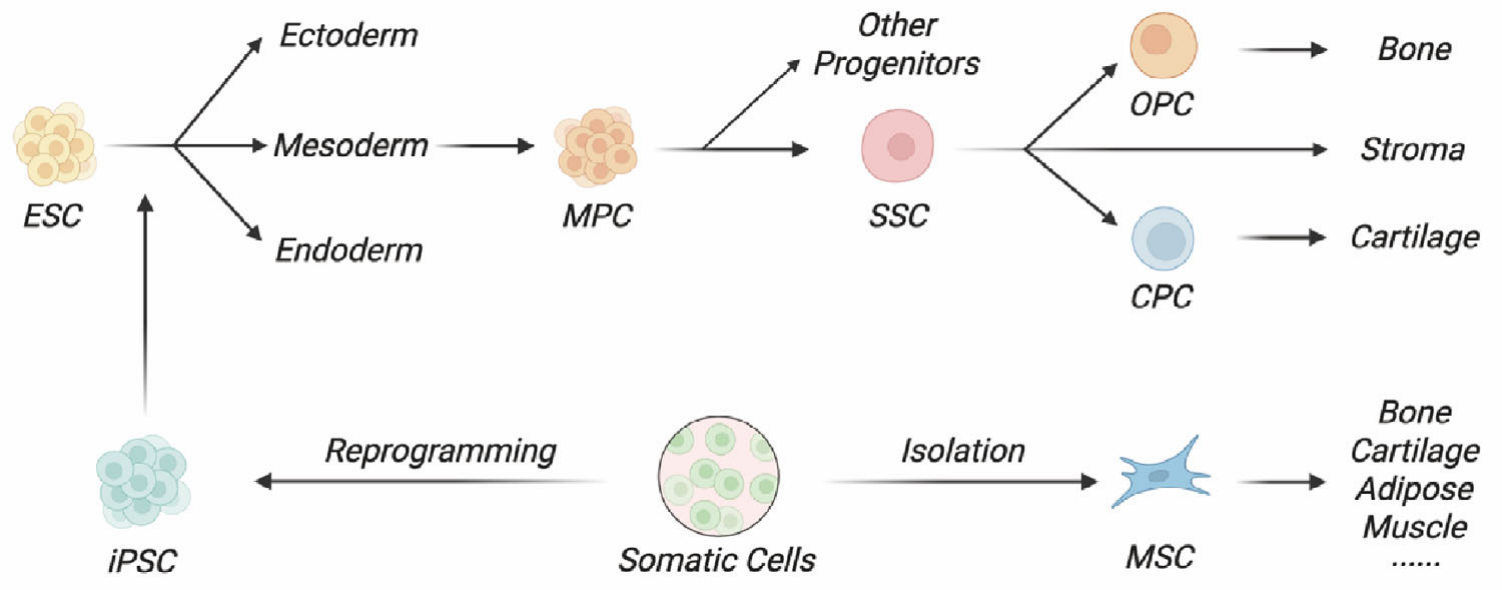
The approximate developmental lineage of stem cells in the skeletal system. ESCs and iPSCs can differentiate into the three germ layers. MPCs, derived from the mesoderm, may further give rise to SSCs. SSCs are a key branching point in skeletal development and can differentiate into cells of the bone, cartilage, and stromal lineages, with OPCs and CPCs serving as the respective progenitors for bone and cartilage. In adult tissues, MSCs can be isolated from somatic cells and possess the potential to differentiate into bone, cartilage, adipose, muscle, and other tissues. The figure was created using BioRender.com.

Ideal seed cells should possess features such as ethical acceptability, accessibility, ease of expansion, batch consistency, and high safety, to maintain their intended lineage fate during in vitro induction and in vivo repair. Although the complete developmental lineage of stem/progenitor cells in the skeletal system has not been fully elucidated, existing studies have revealed the general differentiation trajectory. The prevailing view holds that pluripotent embryonic stem cells (ESCs) first differentiate through a mesenchymal stage into mesenchymal progenitor cells (MPCs), which may subsequently further specialize into progenitors for skeletal development. Recent studies in both mice and human have identified skeletal stem cells (SSCs), a type of tissue‐specific stem cell capable of differentiating into osteocytes, chondrocytes, and stromal cells, but not adipocytes, and they are likely positioned downstream of MPCs. SSCs occupy a critical branching point in skeletal development, and their downstream progeny can progressively differentiate into more tissue‐specific progenitors, such as osteoprogenitor cells (OPCs) and chondroprogenitor cells (CPCs), which are responsible for bone and cartilage formation, respectively (Figure 4) [39, 165, 166, 167, 168, 169, 170, 171]. Although the precise definitions, hierarchical relationships, and surface markers of these cell populations are still being refined, their core roles in embryonic skeletogenesis and adult bone homeostasis have been widely validated. In addition, induced pluripotent stem cells (iPSCs), generated by reprogramming somatic cells, possess pluripotency like ESCs. Considering the ethical and immunological limitations associated with ESCs, iPSCs have become a more feasible alternative [172, 173]. However, their application in BTE remains challenging, as achieving precise and efficient lineage‐specific differentiation control is complex and often inefficient under current strategies. In addition, iPSCs are associated with potential safety concerns, including genetic instability and tumorigenicity, which further limit their direct translational application [174]. Therefore, although iPSCs hold considerable theoretical potential, their application in clinical bone regeneration remains at a developmental stage, and further efforts are needed to optimize differentiation control strategies and improve safety evaluation systems to facilitate their translational use.

In adult tissues, mesenchymal stem/stromal cells (MSCs), distinct from MPCs, do not share a common embryonic origin and do not belong to the same lineage. Adult MSCs generally refer to stem/progenitor cells with a certain capacity for self‐renewal, multipotent differentiation potential (osteogenic, chondrogenic, adipogenic, etc.), and immunomodulatory properties, which can be derived from bone marrow, umbilical cord, adipose tissue, synovium, and other sources [175, 176]. MSCs from different sources show significant differences in their harvesting methods, proliferative capacity, differentiation potential, and immunomodulatory properties (Table 3). Among these, bone marrow‐derived MSCs (BMSCs) were the first widely used in tissue engineering research and remain the most extensively applied, exhibiting strong osteogenic and chondrogenic potential. However, their isolation requires invasive procedures, and they exhibit notable senescence during in vitro expansion. Umbilical cord‐derived MSCs (UCMSCs) can be obtained non‐invasively from umbilical cord tissue, possess high proliferative capacity and strong immunomodulatory properties, and show great preclinical potential, although their chondrogenic potential is generally slightly lower than that of BMSCs. Adipose‐derived MSCs (ADSCs) have advantages due to the abundance and accessibility of adipose tissue, high yield, and strong in vitro expansion capacity, but their chondrogenic potential is generally lower than that of BMSCs and UCMSCs [177, 178, 179]. In addition, synovium‐derived MSCs (SMSCs) are considered to have strong chondrogenic potential, but due to limited studies, their practical applicability in tissue engineering still needs further validation [180]. In addition, dental pulp‐derived MSCs (DPSCs) and placenta‐derived MSCs (PMSCs) have also been reported to possess chondrogenic potential [181, 182]. It should be noted that MSCs from different sources exhibit donor, tissue, and subpopulation heterogeneity, with considerable variability in differentiation capacity between batches, representing a major limitation for their application in tissue engineering and regenerative medicine [183]. Since ECO starts from cartilage and the engineered constructs are intended for in vivo transplantation, the choice of seed cells should consider not only their chondrogenic potential but also their immunogenicity, to better align with natural developmental processes and regenerative logic.

**TABLE 3:** Comparison of MSCs from different sources.

Tissue source	Advantages	Disadvantages	References
BMSC	High osteogenic and good chondrogenic potential	Invasive harvest, donor age affects quality, prone to senescence	[184, 185, 186]
ADSC	Easy to obtain, high proliferation, good immunomodulation	Lower osteogenic and chondrogenic potential	[184, 187]
UCMSC	Young, high immunomodulation, easy harvest, high proliferation	Donar and subpopulation heterogeneity	[188, 189, 190]
SMSC	Excellent cartilage‐specific differentiation	Requires arthroscopic harvest	[191, 192]
DPSC	High osteogenic potential	Prone to form fibrocartilage under chondrogenic induction	[181, 193]
PMSC	Easy harvest, rich in growth factors, high immunomodulatory, strong angiogenic potential	Difficult to purify, low osteogenic potential, tissue‑of‑origin heterogeneity	[194, 195, 196, 197, 198]

Emerging evidence suggests that certain non‐canonical stem/progenitor cell populations derived from other tissues, such as fibro/adipogenic progenitors (FAPs), also participate in the process of endochondral bone repair. FAPs were initially defined in the context of skeletal muscle regeneration and are implicated in fibrosis and adipogenesis. Further studies have demonstrated that FAPs are also involved in heterotopic ossification (HO) [199, 200, 201, 202]. A recent lineage‐tracing study further demonstrates that a PRG4+ FAP subpopulation originating from the skeletal muscle niche can be recruited to the fracture microenvironment, where it responds to local regenerative cues and directly contributes to endochondral bone repair. Notably, the contribution of this cell population is more pronounced in endochondral rather than intramembranous bone repair, suggesting a preferential role of FAPs in ECO [203]. Collectively, these findings further indicate that ideal tissue‐engineering seed cells should not only possess multilineage differentiation potential, but also recapitulate key features of endogenous injury‐responsive progenitors, including microenvironmental adaptability and activation upon injury, as exemplified by the niche‐dependent behavior of FAPs.

Beyond the intrinsic source and characteristics of seed cells, their functions can be further optimized through various molecular and genetic engineering strategies to enhance chondrogenic and osteogenic potential, or to modulate the secretion of bioactive factors, thereby improving the regenerative performance of tissue‐engineered constructs. In theory, regulatory strategies include gene transfection, RNA interference, and genome‐editing tools such as CRISPR/Cas9, among which gene transfection remains the most widely used and mature approach. For instance, studies have shown that overexpression of chondrogenic genes, such as SOX9 or BMP2, in MSCs can significantly enhance cartilage matrix formation in engineered constructs [90], while modulation of key signaling molecules, such as SMAD3 or RUNX2, can precisely direct MSC differentiation toward osteogenic or chondrogenic lineages [25, 204]. In addition, regulating the secretion of pro‐angiogenic factors, such as VEGF, from MSCs can further promote vascularized regeneration [205]. Overall, targeted engineering of seed cells can optimize both the differentiation efficiency of the seed cells and the performance of tissue‐engineered constructs.

In addition, co‐culturing two or more cell types is also an important strategy to enhance the functionality of tissue‐engineered constructs. For example, MSC–Chondrocyte co‐culture can significantly promote the production and deposition of cartilage matrix [206]. Meanwhile, studies have shown that MSC–EC co‐culture combined with the ECO strategy can enhance vascularization while promoting cartilage template formation, thereby accelerating bone formation and remodeling [207, 208]. Another study reported that embedding MSCs and chondrocytes into a bilayer construct, and subjecting MSCs to certain signaling stimuli to undergo ECO while maintaining the chondrocyte phenotype, could achieve bi‐layered osteochondral regeneration [209]. Overall, multicellular co‐culture not only optimizes the microenvironment through direct cell–cell contact and paracrine interactions but also coordinates multiple regenerative pathways, including chondrogenesis, osteogenesis, and angiogenesis, making it a promising approach for constructing fully functional ECO organoids and achieving efficient bone repair.

### 4.2 Biomaterial Scaffolds

ECO tissue engineering can be classified into scaffold‐free and scaffold‐based systems. Scaffold‐free methods mainly include pellet culture, micromass culture, hanging drop, and ultra‐low attachment (ULA) approaches [210, 211]. These strategies share the common feature of relying on tight cell aggregation and self‐assembly under scaffold‐free conditions to form 3D cell aggregates, thereby partially recapitulating the early critical stage of mesenchymal condensation during the embryonic ECO process. As classical and widely applied scaffold‐free approaches, they are relatively simple to perform, cost‐effective, and have therefore been extensively used for in vitro cartilage and ECO‐related model construction. However, their limitations lie in the suboptimal structural stability and size uniformity of the resulting cell aggregates, making them unsuitable for high‐throughput standardized fabrication. In addition, due to the lack of exogenous structural support, they exhibit limited mechanical strength and are prone to diffusion constraints, which further restrict their application in constructing large‐scale cartilage or bone organoids and in long‐term culture.

Compared with scaffold‐free systems, scaffold‐based systems provide a stable 3D structural framework that supports cell adhesion, proliferation, and differentiation, which is particularly beneficial for the formation of larger and more complex tissue constructs. It is generally recognized that scaffold‐based approaches remain the dominant strategy for achieving mechanical integrity and spatial guidance in cartilage and bone tissue engineering. In addition, scaffolds can recapitulate key features of the native ECM microenvironment and can be further engineered through biochemical modification and mechanical tuning to precisely regulate cell behavior and tissue organization [212, 213, 214, 215]. Based on their source and properties, scaffold materials commonly used in BTE can be classified into four categories: natural polymers, synthetic polymers, inorganic materials, and decellularized ECM (dECM) (Table 4) [216, 217, 218, 219, 220, 221, 222]. For example, collagen has been extensively demonstrated as an effective material for cartilage constructs, capable of faithfully mimicking the developmental pattern of ECO [20, 223, 224]; biomimetic dECM derived from cartilage (or hypertrophic cartilage) and bone can preserve the bioactivity of the matrix and recapitulate the ECO process of stem cells to induce bone formation [25, 35, 225].

**TABLE 4:** Scaffold materials for ECO‐based engineering strategies.

Category	Representative materials	Advantages	Limitations
Natural polymers	collagen, gelatin/GelMA, hyaluronic acid (HA), fibrin, silk fibroin	High biocompatibility; strong cell adhesion; ECM‐mimicking bioactivity	Weak mechanical strength; fast degradation; poor processability
Synthetic polymers	PCL, PLA, PLGA, PEG‐based hydrogels	Tunable mechanical properties; controllable degradation; good processability	Limited bioactivity; poor cell recognition; potential cytotoxicity and inflammatory responses from degradation products
Inorganic materials	hydroxyapatite (HA), β‐tricalcium phosphate (β‐TCP), bioactive glass	Strong osteoinductive potential; high osteoconductivity; good mechanical strength	Brittleness; limited elasticity; poor intrinsic bioactivity; require composite design for functional tuning
decellularized ECM (dECM)	cartilage dECM, hypertrophic cartilage dECM, bone dECM	Native biochemical cues; preserved microarchitecture; strong bioactivity	Batch‐to‐batch variability; uncontrolled degradation; complex composition and unclear mechanism of action

Given the complexity and staged nature of the ECO process, effective scaffold design in cartilage‐bone tissue engineering must comprehensively account for multiple factors. First, scaffolds should possess appropriate pore structure and interconnectivity to support cell adhesion, migration, and 3D diffusion, while facilitating the transport of nutrients, oxygen, and metabolic waste, which also promotes vascularization and new bone formation. Optimizing pore size is particularly critical: studies have shown that smaller pores favor cartilage formation, whereas larger pores (>300 µm) support vascularization and osteogenesis (though this depends on the material type). Thus, scaffold design must balance the requirements for both cartilage and bone formation while considering mechanical performance [226, 227, 228, 229]. Second, the mechanical properties of the scaffold should match those of the target tissue to avoid stress shielding from excessive stiffness or insufficient support from overly soft scaffolds. Research indicates that softer matrices favor cartilage formation, whereas stiffer matrices support osteogenesis, which helps explain why natural polymers such as collagen are more suitable for ECO engineering than comparatively rigid inorganic materials [230, 231]. Moreover, the elastic properties of scaffolds also influence cartilage formation, with matrices exhibiting rapid stress relaxation being more conducive to chondrogenesis [232]. Third, scaffold degradability should be synchronized with tissue formation, allowing the scaffold to provide initial structural support while gradually being replaced by newly formed tissue, thereby preventing premature collapse or long‐term persistence that could hinder tissue integration [233]. Finally, scaffolds should possess bioactivity and good biocompatibility to minimize toxicity, inflammation, and immune rejection [234]. Additionally, scaffolds can be designed to be environmentally responsive, dynamically adjusting their structure, degradability, and bioactive factor release in response to changes in pH, temperature, or enzymatic activity, further enhancing their functionality and adaptability in vivo [235, 236]. By integrating considerations of pore structure/size, mechanical property, degradability, and biocompatibility, scaffolds can provide a platform for endochondral bone formation that is both functional and safe.

However, despite these advantages, scaffold‐based systems still face several intrinsic limitations that hinder their faithful recapitulation of native tissue development and full clinical translation. One major challenge is the lack of sufficient control over spatial and temporal heterogeneity within the scaffold microenvironment, which limits the ability to fully mimic the dynamic and region‐specific cues present in native tissues [27, 237]. In addition, many conventional scaffold materials exhibit suboptimal integration with host tissues, leading to issues such as poor vascularization, delayed remodeling, or fibrotic encapsulation after implantation [238, 239, 240]. Furthermore, the fabrication of highly complex, hierarchical structures often relies on trade‐offs between mechanical strength, porosity, and biological functionality, making it difficult to simultaneously achieve all desired properties within a single scaffold system. Another critical limitation lies in the batch‐to‐batch variability and limited reproducibility associated with natural or ECM‐derived materials, which can compromise experimental consistency and translational reliability. Collectively, these challenges highlight the need for more advanced scaffold design strategies capable of integrating dynamic regulation, multiscale structural control, and enhanced biological fidelity.

In recent years, advances in biomaterials engineering have significantly expanded the design concepts and functional boundaries of scaffold‐based systems. In particular, several major emerging directions have attracted considerable attention. These include 3D bioprinting and related additive manufacturing technologies, which enable precise spatial construction and multi‐material integration [241, 242]; microfluidic technologies enabling precise control of microscale tissue formation and high‐throughput tissue assembly, together with organ‐on‐a‐chip (OOAC) systems recapitulating organ‐level physiology under dynamic perfusion, represent key advances in engineered tissue modeling [237, 243, 244, 245, 246]; stimuli‐responsive hydrogel systems, which can dynamically and programmably regulate material properties in response to external environmental cues [247, 248]; and self‐assembly strategies, which enable biomaterials and cells to autonomously organize into hierarchically structured constructs without external scaffolds [249, 250]. Collectively, these approaches have enhanced the structural complexity, spatial controllability, and functional adaptability of engineered constructs. In addition, tissue‐specific dECM scaffolds have emerged as important biomimetic platforms, preserving the biochemical composition and microenvironmental cues of native tissues and demonstrating unique advantages in promoting cell–matrix interactions and tissue‐specific regeneration. Overall, these advances reflect a growing consensus that the integration of structural precision, dynamic adaptability, and biological fidelity is essential for recapitulating developmental microenvironments and achieving effective tissue engineering‐based repair, and they are expected to be further applied to the design and construction of scaffolds for ECO‐related tissue engineering.

### 4.3 Bioactive Factors

Bioactive factors are key exogenous molecules that regulate the differentiation of seed cells and can guide tissue formation at different stages of ECO. They promote chondrogenesis, hypertrophy, and subsequent ossification, while also supporting extracellular matrix deposition, vascularization, and mineralization. Based on the current understanding of ECO biological processes and the regulation of key signaling molecules, commonly used induction schemes include: (1) chondrogenic stage, factors that promote cartilage formation such as BMP, TGF‐β, GDF5 (growth differentiation factor 5), ascorbic acid (Vit C), and KGN (Kartogenin); (2) angiogenic stage, factors that promote vascularization such as VEGF, HIF‐1α, bFGF, IGF‐1, and PDGF; and (3) osteogenic stage, factors that promote bone formation such as BMP and thyroid hormone (T3) [20, 251, 252, 253, 254]. In recent years, new molecules and compounds have continuously been discovered [255, 256, 257]. These factors can not only serve as exogenous inducers but can also be integrated into biomaterial scaffolds, allowing precise regulation of cell behavior and tissue formation through controlled release or genetic engineering strategies.

The integration of scaffolds with bioactive factors is an effective strategy to precisely regulate cell differentiation and tissue formation. Beyond conventional strategies such as physical adsorption and chemical crosslinking, recent advances in micro‐ and nanoscale carrier systems have further improved the spatiotemporal control of factor delivery. In particular, biodegradable polymer‐based microspheres and nanoparticles, such as PLGA systems, enable sustained and localized release of growth factors. Their release kinetics can be finely tuned by adjusting polymer composition, degradation rate, and particle architecture, thereby allowing precise modulation of dose, duration, and biological activity within the target microenvironment. Encapsulation of bioactive factors within these carriers, followed by their incorporation into scaffolds, further enables controlled spatial and temporal release, including staged multi‐factor delivery [258, 259, 260]. In addition, gradient‐functionalized scaffolds can be designed to establish spatial gradients or region‐specific distributions of bioactive factors. Such spatial control can be achieved by regulating the deposition of growth factor‐loaded particles or constructing aligned fibrous architectures, thereby mimicking in vivo signaling landscapes and potentially guiding the stratified formation of cartilage and bone [28, 33, 35, 209, 261, 262]. At the structural engineering level, coaxial electrospun nanofibrous scaffolds with core–shell architectures enable sequential and temporally programmed release of multiple bioactive signals, such as the rapid release of chemotactic factors (e.g., SDF‐1) from the shell and sustained release of osteoinductive factors from the core, thereby coordinating cell recruitment and differentiation [263]. Beyond passive delivery systems, stimuli‐responsive smart biomaterials have emerged as a key strategy for dynamic and on‐demand regulation of factor presentation, enabling localized release through conformational changes or bond cleavage in response to endogenous or exogenous stimuli such as pH, enzymatic activity, temperature, redox state, or light, thereby dynamically regulating factor release in response to changes in the microenvironment and further enhancing in vivo functionality and adaptability [264, 265, 266]. In addition, engineered affinity components, such as heparin‐based delivery platforms, enhance the stability of specific growth factors, reduce their degradation, and enable controlled release through affinity interactions [267, 268]. A rational integration of these strategies helps establish a highly coordinated scaffold–factor system, enabling precise spatiotemporal regulation of cell behavior and tissue morphogenesis.

**FIGURE 5:**
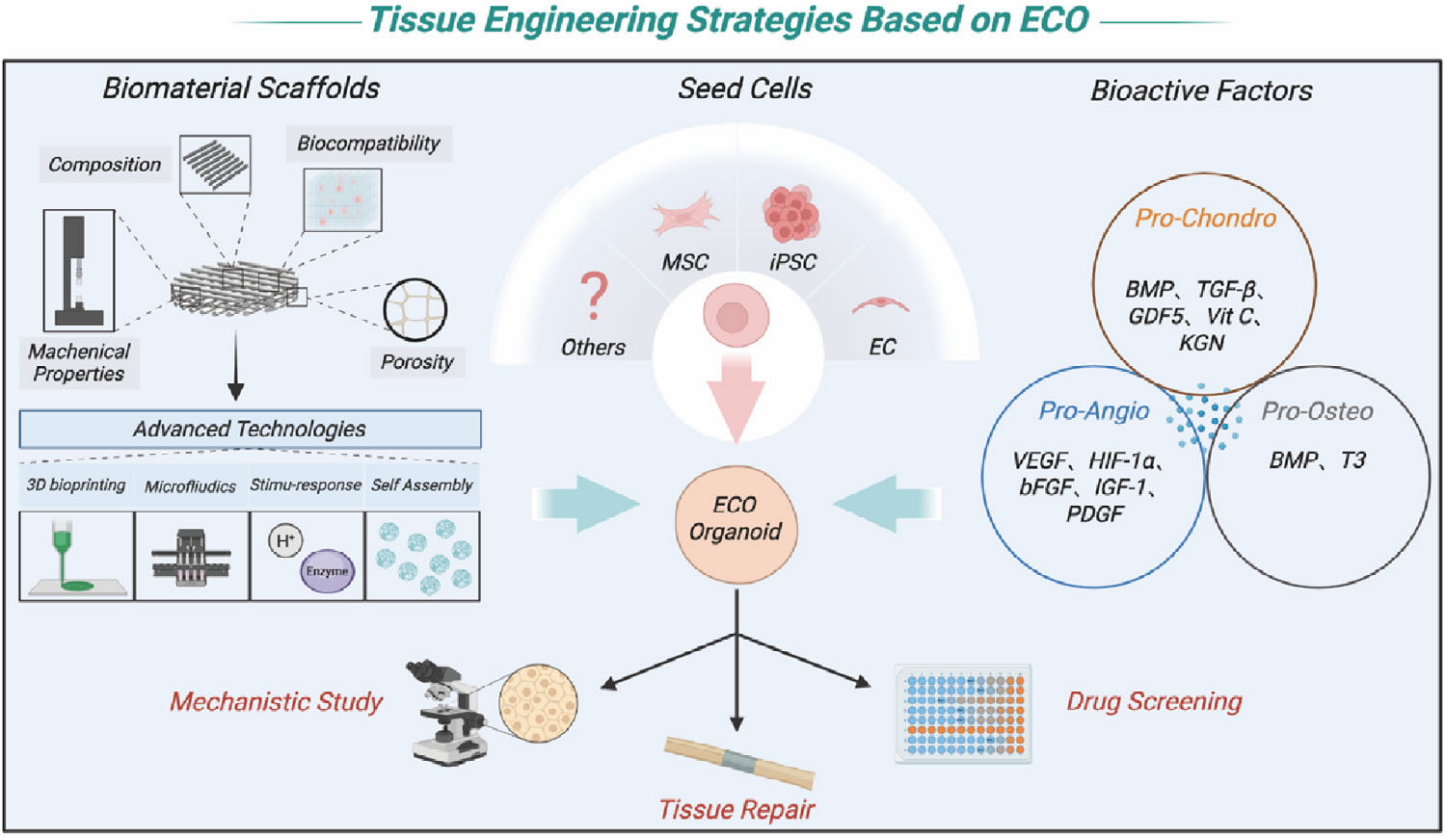
Tissue engineering strategies based on ECO. ECO organoids are constructed around three fundamental components—seed cells, engineering scaffolds, and bioactive factors—to recapitulate the developmental progression from cartilage to bone, offering broad research and translational potential. Their primary applications include: (1) serving as tissue‐engineered grafts for repairing large bone defects; (2) enabling in vitro studies to elucidate cell fate transitions, signaling pathways, and regulatory mechanisms during the ECO process; and (3) facilitating drug screening for agents that promote chondrogenesis or osteogenesis. The figure was created using BioRender.com.

Overall, the synergistic integration of seed cells, biomaterial scaffolds, and bioactive factors constitutes the three core elements for constructing highly biomimetic and functional “ECO organoids” (Figure 5). Seed cells provide regenerative potential, scaffolds offer 3D structure and mechanical support, and bioactive factors precisely regulate stem cell behavior and lineage differentiation. Through their interactions and signal amplification, combined with spatial design and temporal control of signaling, this integration can significantly enhance the efficiency and quality of tissue regeneration. On one hand, this integrated strategy services as tissue‐engineered grafts for large bone defects. On the other hand, it also provides a reliable in vitro platform that facilitates the elucidation of cell fate transitions, signaling pathway regulation, and tissue remodeling during skeletal development, while serving as an efficient system for screening and evaluating novel therapeutics for related diseases, thereby bridging regenerative medicine, basic research, and drug development.

### 4.4 Common Organoid Construction Strategies Based on ECO

Organoids are 3D self‐organizing structures that recapitulate key features of native tissues, providing a powerful platform for developmental studies and disease modeling. Current tissue engineering strategies emphasize the recreation of functional tissues by mimicking developmental and regenerative processes, whereas organoid technology relies on the assembly and differentiation of stem cells to reconstruct endogenous developmental programs, providing a rationale for its application in BTE. By constructing appropriate organoids, the stage‐wise progression of ECO can be recapitulated in vitro, thereby providing an effective model for investigating bone developmental mechanisms and optimizing regenerative strategies.

In vitro construction of organoids employs various tissue engineering strategies to recapitulate bone and cartilage formation and promote tissue regeneration. Generally, these strategies follow an initial chondrogenic phase, which is subsequently followed by hypertrophy and ossification. For instance, in both scaffold‐based and scaffold‐free systems, seed cells such as MSCs and iPSCs are typically induced toward chondrogenic differentiation for 3–4 weeks to form 3D spheroids resembling hyaline cartilage, which are then subjected to 1–2 weeks of hypertrophic induction to generate “bone organoids” with mineralized bone tissue [20, 269]. Based on this approach, ECO‐based organoids currently include callus organoids, ECO organoids, bilayer osteochondral organoids, and osteoarthritis (OA) organoids (Table 5). Callus organoids are primarily used to obtain cartilage intermediates suitable for in vivo implantation [22, 270], while ECO organoids recapitulate the full trajectory from cartilage to bone in vitro [21, 223, 271]. Bilayer osteochondral organoids typically combine hyaline cartilage formation and ECO strategies to repair articular osteochondral defects [28, 35, 209]. OA organoids, on the other hand, utilize the ECO strategy to mimic cartilage degeneration and ossification during pathological processes, serving as disease models and platforms for drug screening, thereby demonstrating considerable translational potential [252]. Notably, recent studies have even successfully generated bone organoids with an autonomous hematopoietic niche in vitro using iPSCs, marking a historic breakthrough in organoid vascularization and opening new avenues for bone tissue engineering and regenerative medicine research.

**TABLE 5:** Organoid construction strategies based on ECO.

	Seed cells	Scaffold	Differentiation strategy	Special regulation	Reference
Callus Organoids	Equine BMSCs	GelMA	2W chondrogenesis→subcutaneous implantation	embedded with cartilage‐derived matrix (CDM) particles	Visser et al., 2015 [272]
	hBMSCs + HUVECs	PCL nanofiber mesh	MSC chondrogenesis for 3W→co‐cultured with HUVECs for 3W→subcutaneous implantation	—	Freeman et al., 2015 [208]
	hPDCs	—	3W chondrogenesis→assembly→subcutaneous implantation	—	Nilsson Hall et al., 2019 [22]
	hBMSCs	GelMA	3W chondrogenesis→subcutaneous implantation	—	Xie et al., 2022 [270]
	hPDCs	—	3W chondrogenesis→assembly→subcutaneous implantation	—	Decoene et al., 2025 [273]
ECO Organoids	hBMSCs	—	3W chondrogenesis →2W hypertrophy	—	Scotti et al., 2010 [21]
	hBMSCs	COL‐I	3W chondrogenesis→2W hypertrophy	—	Scotti et al., 2013 [20]
	hADSCs	Decellularized bone scaffold	2W chondrogenesis→3W hypertrophy	—	Bernhard et al., 2017 [225]
	human adipose tissue	—	4W chondrogenesis→2W hypertrophy	—	Guerrero et al., 2018 [271]
	iPSCs	—	Mesoderm induction→2W chondrogenesis→2W hypertrophy	3D suspension culture	Zhang et al., 2020 [251]
	Human and rat BMSCs	Collagen hydrogel	3W chondrogenesis→10d hypertrophy→devitalization→implantation subcutaneously or into a bone defect	—	Longoni et al., 2022 [274]
	hUCMSC	COL‐I	4W chondrogenesis→2W hypertrophy	Exendin‐4 stable transfection	He et al., 2023 [223]
	hEPSCs	—	4W chondrogenesis→2W hypertrophy	COL2A1‐mCherry and COL10A1‐eGFP double reporter	Wei et al., 2024 [269]
Bilayer Osteochondral Organoids	Porcine Chondrocytes + MSCs	Bi‐Layered hydrogel	3W chondrogenesis→4W hypertrophy	—	Sheehy et al., 2013 [275]
	Human nasal chondrocytes + BMSCs	Bi‐layered hydrogels	Subcutaneous implantation without pre‐differentiation	Layered biotinylated BMP‐2 or TGF‐β3	Stüdle et al., 2018 [209]
	Rabbit BMSCs	3D bio‐printed anisotropic scaffold	8W chondrogenesis→ implantation into a bone defect	PTH (outer)/HA (inner) + mechanical stimulation	Li et al., 2023 [33]
OA Organoids	hBMSCs	—	2W chondrogenic→2W hypertrophic and pro‐inflammatory	—	Dönges et al., 2024 [252]

Although human organoid‐based approaches hold conceptual promise in BTE, it is important to acknowledge their current translational limitations. While ECO‐related and other bone organoid systems can successfully recapitulate key developmental features in vitro, their application in achieving clinically meaningful bone regeneration remains limited. In particular, most existing systems have not yet demonstrated robust vascular integration, sufficient biomimetic fidelity, or the scalability and standardization required for clinical translation [276, 277]. Therefore, current organoid models are largely restricted to in vitro developmental studies, disease modeling, or proof‐of‐concept regenerative strategies, rather than established therapeutic approaches for bone defect repair. This limitation highlights the substantial gap that still exists between organoid‐based modeling and clinically effective bone regenerative therapies.

## 5 Advancing Future Optimization Strategies

**FIGURE 6:**
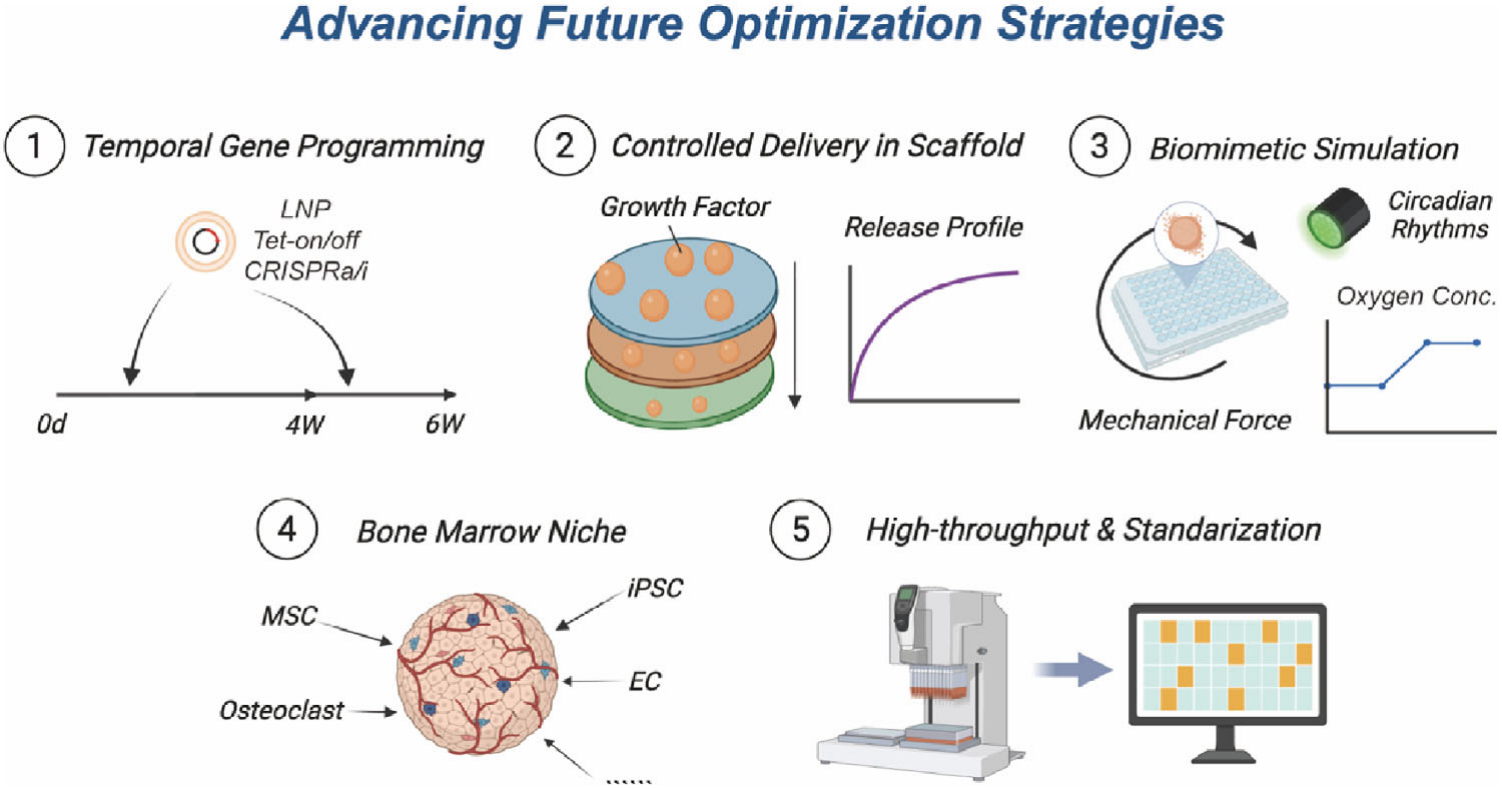
Potential optimization directions for future ECO engineering strategies. (1) Temporal gene expression programming for stem cells. (2) Incorporation and controlled release of bioactive factors in scaffolds. (3) Biomimetic simulation of exogenous physical and chemical factors. (4) Engineering vascularized organoids with functional bone marrow niches. (5) Standardized and high‐throughput engineering technologies. The figure was created using BioRender.com.

We propose that, to further enhance the engineering and translational potential of ECO strategies in bone repair, future optimization should be pursued from multiple perspectives. Specifically, we suggest focusing on temporal control of stem cell differentiation, the introduction and controlled release of bioactive factors, biomimetic simulation of exogenous physical and chemical cues, construction of functional bone marrow microenvironments, and the implementation of standardized and high‑throughput engineering technologies (Figure 6). Overall, these strategies are expected to provide both theoretical and technical support for the development of more precise, functional, and scalable ECO engineering approaches.

### 5.1 Temporal Gene Expression Programming for Stem Cells

In current strategies, stem cells typically rely on exogenous inductive factors to achieve directed differentiation, such as TGF‐β, BMPs, and pro‐hypertrophic signals. In addition, intrinsic regulation of gene expression within the seed cells has also been explored, including stable transfection of some genes (e.g., BMP2) to enhance differentiation efficiency [25, 223]. However, conventional programming approaches are predominantly based on permanent gene editing, and most inductive factors are unable to simultaneously promote both chondrogenic and osteogenic processes, which are stage‐specific and require distinct cues. As a result, these strategies are insufficient to accommodate the multi‐stage differentiation process of ECO. If gene expression remains fixed over time, it may lead to asynchronous differentiation, signaling disruption, or premature progression into the osteogenic phase, ultimately compromising organoid functionality and bone regeneration outcomes.

To address these limitations, future strategies should not only focus on optimizing the spatiotemporal presentation of bioactive factors within scaffolds, but also incorporate exogenous gene regulation tools to achieve more precise control over stem cell differentiation. Among these, RNA‐based delivery systems, particularly lipid nanoparticles (LNPs), have emerged as a powerful platform for modulating cellular behavior due to their efficient nucleic acid delivery, low toxicity, and favorable biocompatibility. Compared with conventional stable transfection methods, LNP‐mRNA delivery systems enable non‐integrating, reversible, and temporally controllable gene expression, thereby avoiding potential differentiation disorders caused by prolonged or constitutive expression of exogenous genes. In addition, relative to directly adding growth factors to induction media, LNP‐mediated delivery can enhance cellular uptake and prolong the effective duration [278, 279, 280]. Importantly, such systems are particularly advantageous for in vivo applications in BTE. For example, an LNP‐based mRNA therapeutic encoding β‐catenin was developed to activate the Wnt/β‐catenin signaling pathway, and local delivery significantly enhanced bone formation and accelerated cartilage‐to‐bone transformation in a murine fracture model [281]. Similarly, another study demonstrated that delivery of chemically modified BMP‐2 mRNA promotes bone regeneration in large segmental defects via endochondral bone repair, improves mechanical recovery, and shows potential as an alternative to conventional rhBMP‐2 therapy [282]. Collectively, these studies highlight the potential of LNP‐mRNA therapeutics as a versatile and translatable platform for achieving controlled, stage‐specific gene regulation in bone regeneration.

Moreover, inducible or self‐regulating gene expression systems, such as Tet‐On/Tet‐Off and CRISPRa/i, can precisely modulate exogenous or endogenous gene expression at predetermined time points, enabling more flexible temporal control [283, 284, 285]. These approaches provide more programmable and sustained regulation of gene networks, making them particularly suitable for coordinating the sequential differentiation processes required during ECO. For instance, doxycycline‐inducible CRISPRa/i systems in MSCs have been used to reversibly regulate osteogenic genes such as ALP, thereby modulating osteogenic differentiation [286]; in addition, CRISPRai‐mediated activation of SOX9 combined with repression of PPARγ has been shown to promote chondrogenesis and improve calvarial bone healing [287]. Integrating these strategies enables on‐demand and stage‐specific regulation of gene expression, thereby facilitating the coordinated and hierarchical differentiation of cells during ECO.

### 5.2 Incorporation and Controlled Release of Bioactive Factors in Scaffolds

Modification and controlled release of bioactive factors within scaffolds are critical for regulating stem cell behavior; however, current material designs remain limited in their ability to fully recapitulate the dynamic and hierarchical signaling environment of ECO. Most conventional scaffolds provide uniform loading and fixed release profiles, which fail to reproduce the spatial and temporal gradients of growth factors observed in native bone development. In layered structures such as articular cartilage, factors like VEGF, BMPs, and TGF‐β exhibit concentration gradients across different depths, and these spatial gradients play a crucial role in guiding the “polarity” of cellular differentiation [107, 288, 289]. In addition, the biological activity of incorporated factors may be compromised during release or due to unstable interactions with scaffold matrices, further limiting therapeutic efficacy.

To address these limitations, recent advances in scaffold engineering have focused on improving both spatial patterning and temporal control of bioactive cues. Spatially, microfabrication strategies such as microfluidics and advanced 3D printing have been used to construct gradient scaffolds that better mimic native morphogen distributions, thereby guiding region‐specific stem cell differentiation [246]. For example, a gradient hydrogel with bioactive glass has been developed to recapitulate the native tendon–bone interface microenvironment, where spatially controlled ion release directs BMSCs toward osteogenic differentiation in high‐concentration regions and chondrogenic differentiation in low‐concentration regions, thereby significantly promoting the synchronized regeneration of tendon, fibrocartilage, and bone at the enthesis [290]. Temporally, degradation‐tunable carriers, including polymeric microspheres and nanoparticulate systems, enable staged release of multiple signals to sequentially regulate chondrogenic, angiogenic, and osteogenic events. For example, dual‐factor delivery systems incorporating TGF‐β and BMP‐2 have been shown to recapitulate key stages of ECO by promoting early cartilage formation followed by later mineralization and bone maturation [291]. Importantly, these scaffold‐based strategies have been validated in preclinical bone regeneration models, including critical‐sized defect and fracture repair settings. Spatiotemporally controlled delivery of inductive factors has been shown to significantly enhance vascularized bone formation and functional tissue reconstruction. Although scaffold systems are effective in organizing biochemical signals in both space and time, they still lack adaptive responsiveness to the evolving states of cells. This highlights the need for next‐generation biomaterials capable of dynamically interacting with the biological microenvironment during regeneration.

### 5.3 Biomimetic Simulation of Exogenous Physical and Chemical Factors

Although current induction systems for engineered constructs have become increasingly sophisticated, they still fail to fully recapitulate the highly dynamic and spatiotemporally regulated microenvironment of native endochondral bone development. From the perspective of biomimetic simulation of exogenous physical and chemical cues, in vivo ECO is orchestrated by a tightly coordinated interplay between biochemical signals and physicochemical stimuli that collectively regulate tissue formation, maturation, and vascular invasion. Among these regulatory inputs, mechanical stimulation, oxygen tension, and circadian rhythm–associated temporal regulation represent three key yet often underappreciated dimensions for enhancing the fidelity of engineered ECO systems.

Mechanical stimulation, such as fluid shear stress generated by rotating culture and cyclic tensile strain, can promote cartilage ECM deposition [251, 292, 293], although further studies are needed to fully validate these effects. For example, bioreactor systems can be designed to recapitulate the joint cavity–associated mechanical microenvironment, thereby not only promoting the formation and maturation of cartilage but also serving as standardized platforms for the screening and functional evaluation of engineered cartilage constructs [294, 295]. In the future, bioreactor systems are expected to enable precise and adaptive regulation of dynamic and multi‐modal mechanical parameters, including magnitude, frequency, waveform, and spatial distribution, through programmable and feedback‐controlled designs, thereby more closely mimicking the in vivo developmental mechanical niche.

Oxygen gradients are also critical in ECO: early hypoxia can promote hyaline cartilage differentiation via HIF‐1α–mediated upregulation of SOX9 and downregulation or RUNX2, while elevated oxygen levels at later stages favor osteogenesis and mineralization [296, 297, 298]. Temporal precision can be achieved through exogenous modulation of oxygen concentration or the use of oxygen‐responsive materials, thereby facilitating sequential stem cell differentiation. For example, oxygen tension–regulating hydrogels have been engineered through staged release of hypoxia‐inducing copper ions and oxygen‐generating calcium peroxide, enabling sequential activation and deactivation of HIF‐1α signaling and thereby coordinating angiogenic and osteogenic transitions during bone regeneration [299]. In addition, hypoxia‐mimicking 3D biomineralized hydrogel systems integrated with structural scaffolds have been shown to recapitulate the early hypoxic chondrogenic microenvironment while simultaneously supporting later‐stage osteogenic mineralization [31], further demonstrating the feasibility of oxygen‐guided strategies for endochondral bone regeneration. From an engineering perspective, such stage‐dependent oxygen regulation can be achieved using gas‐perfusion bioreactors, oxygen‐permeable materials, or oxygen‐releasing and ‐consuming systems, enabling the establishment of programmable oxygen gradients that guide continuous cell fate transitions during ECO.

Moreover, inspired by the circadian regulation of long bone growth during adolescence, emerging evidence suggests that ECO progression may also be influenced by intrinsic circadian rhythms, particularly through core clock regulators such as BMAL1 (brain and muscle ARNT‐like 1) [300, 301, 302]. Based on this understanding, at the engineering level, circadian entrainment can be partially modulated using external timing cues, such as light–dark cycles or temporal delivery of clock‐associated biochemical factors, although precise genetic‐level control of core clock genes remains technically challenging in current organoid systems and largely unexplored in the context of ECO engineering. Integrating circadian synchronization strategies into ECO‐based organoid engineering may help better align in vitro development of engineered constructs with in vivo physiological rhythms, thereby improving tissue maturation and functional fidelity, and represents a promising direction for future bioengineering development.

### 5.4 Engineering Vascularized Organoids With Functional Bone Marrow Niches

In vitro vascularization remains a central bottleneck in bone organoid engineering and a key determinant of both regenerative efficacy and clinical translational potential. Although human vessel organoids have been successfully established [303, 304], current bone organoid systems still rely heavily on pro‐angiogenic factors or the incorporation of endothelial cells (ECs) to enhance vasculogenic capacity, yet fail to form fully functional vascular networks in vitro [207, 208, 305]. Moreover, even after implantation, vascularization within engineered constructs is often incomplete or poorly organized, limiting efficient integration with host vasculature and perfusion. In addition, the absence of a functional bone marrow niche with hematopoietic activity further restricts the physiological relevance of these systems, as vascularization and marrow function are tightly coupled in native bone, collectively highlighting a critical barrier to the development of fully biomimetic bone organoids.

To address the critical bottleneck of organoid vascularization, systematic optimization is required across cellular composition, spatial architecture, and microenvironmental signaling. At the cellular level, engineering strategies have increasingly focused on reconstructing a multilineage microenvironment that more closely resembles the native bone marrow niche. Beyond conventional MSC‐based systems, iPSCs with higher developmental potential can be induced toward endothelial lineages [306, 307] and integrated with pre‐differentiated cartilage or bone constructs to enhance the vascularization capacity of organoids. Meanwhile, the incorporation of hematopoietic stem/progenitor cells (HSPCs) enables partial reconstitution of hematopoietic functionality [308]. Considering that current in vitro ECO engineering systems still struggle to recapitulate both osteogenesis and bone resorption, the inclusion of osteoclast (precursor) cells derived from bone marrow macrophage lineages is also essential. Recently, attempts have been reported in which human monocyte‑derived osteoclasts were incorporated into 3D in vitro ECO models to mimic osteoclast‑mediated cartilage matrix resorption and infiltration into mineralized regions, thereby enhancing the physiological relevance of the system [309]. Overall, these strategies highlight the importance of coordinating multilineage stem/progenitor cells to establish a functional and self‐sustaining bone marrow niche in vitro, with the aim of achieving improved post‐implantation vascularization and osteogenesis in vivo.

In addition, at the structural and microenvironmental level, hierarchical scaffolds and microcavity architectures can be designed to recapitulate the spatial organization of trabecular bone, vascular networks, and marrow cavities, thereby promoting the formation of characteristic type H vessels and enabling more physiological osteogenesis–angiogenesis coupling [310, 311, 312]. Furthermore, scaffold surfaces can be functionalized with angiogenic factors (e.g., VEGF, HIF‐1α) and hematopoietic‐supporting cues (e.g., SCF, CXCL12) [313] to establish localized signaling gradients that enhance both vascularization and hematopoietic support. The integration of spatially organized architectures with biomolecular signaling cues provides a synergistic engineering framework for reconstructing a functional vascular–marrow microenvironment. Collectively, these strategies hold promise for enabling the development of advanced vascularized organoids with functional bone marrow niches. A recent study has further demonstrated this multi‐dimensional engineering principle in engineered vascularized osteoblastic niche (eVON) systems, which integrate iPSC‐derived cellular components, hydroxyapatite‐based scaffolds, and niche‐associated signaling cues to recapitulate endosteal bone marrow microenvironments and support HSPC maintenance as well as multilineage hematopoiesis in vitro and in vivo [314], representing a paradigm of integrated system engineering.

### 5.5 Standardized and High‐Throughput Engineering Technologies

Currently, the in vitro construction of ECO organoids still relies heavily on manual operations and is highly sensitive to specific experimental conditions, resulting in significant batch‐to‐batch variability, poor reproducibility, and limited scalability. These limitations not only restrict their widespread application in basic research but also impede their translational potential in regenerative medicine. Therefore, future development should prioritize the establishment of standardized, automated, and high‐throughput engineering platforms for ECO organoid fabrication.

To achieve this goal, a hierarchical optimization strategy is required across cellular, process, and system levels. At the cellular level, unification of cell sources is essential to ensure consistent proliferative capacity, differentiation potential, and signaling responsiveness. Notably, certain stem cell populations, such as iPSCs and UCMSCs, which have demonstrated safety and functional stability in clinical studies, may serve as standardized cellular building blocks for organoid engineering [315, 316, 317, 318]. At the process level, the development of standardized and scalable culture systems is critical. Microfluidic platforms, porous microreactors, and automated liquid‐handling systems can be leveraged to precisely regulate key microenvironmental parameters, including nutrient delivery, oxygen tension, and fluid shear stress, thereby enabling high‐throughput parallelized culture and minimizing operator‐induced variability. At the system level, integrated intelligent monitoring and quality‐control platforms are required to dynamically track organoid growth, differentiation status, extracellular matrix deposition, and lineage‐specific gene expression. Such systems, often based on reporter constructs and functional readouts, can enable automated selection or exclusion of organoids that fail to meet predefined quality thresholds [319, 320].

Through the integration of standardized cellular resources, automated culture processes, and intelligent quality‐control systems, a fully controllable and scalable ECO organoid engineering platform is expected to be established, providing a robust technical foundation for regenerative medicine and translational applications.

## 6 Research Trends in ECO for Bone Repair

To systematically reveal the developmental landscape and research hotspots of ECO in bone repair, we conducted a bibliometric and visual analysis of related studies from 1996 to 2025. Overall, both the volume of publications and research interest in ECO have shown a steady increase worldwide. Keyword analysis further suggests that the field is gradually shifting from mechanism‐oriented studies toward more application‐driven, engineering‐focused, and translational research.

Data Source and Search Strategies: All literature were collated from the Science Citation Index (SCI) Expanded database of the Web of Science Core Collection (WoSCC). The search terms were as follows: Topic = (endochondral ossification or endochondral bone formation) AND Topic = (bone repair or bone regeneration) AND Year Published = (1996‐2025) AND Document Type = (Article or Review) AND Language = (English). The inclusion criteria are as follows: (1) research focuses primarily on ECO in bone repair; (2) document types must be article or review; (3) publications must be written in English; (4) publication dates must be between 1996 and 2025. And exclusion criteria are as follows: (1) published topics unrelated to ECO in bone repair; (2) documents such as proceeding paper, book chapters, retracted publication and so on. All valid data from the literature were saved in the format of a download.txt file and subsequently imported into Excel 2021. Co‐authors (YQS and ZHH) independently retrieved and extracted all data. In case of discrepancies, resolutions were reached through expert consultation to achieve a final consensus. A bibliometric and visualized analysis was performed using GraphPad Prism 9, VOSviewer 1.6.20 and CiteSpace 6.2. R4.

**FIGURE 7:**
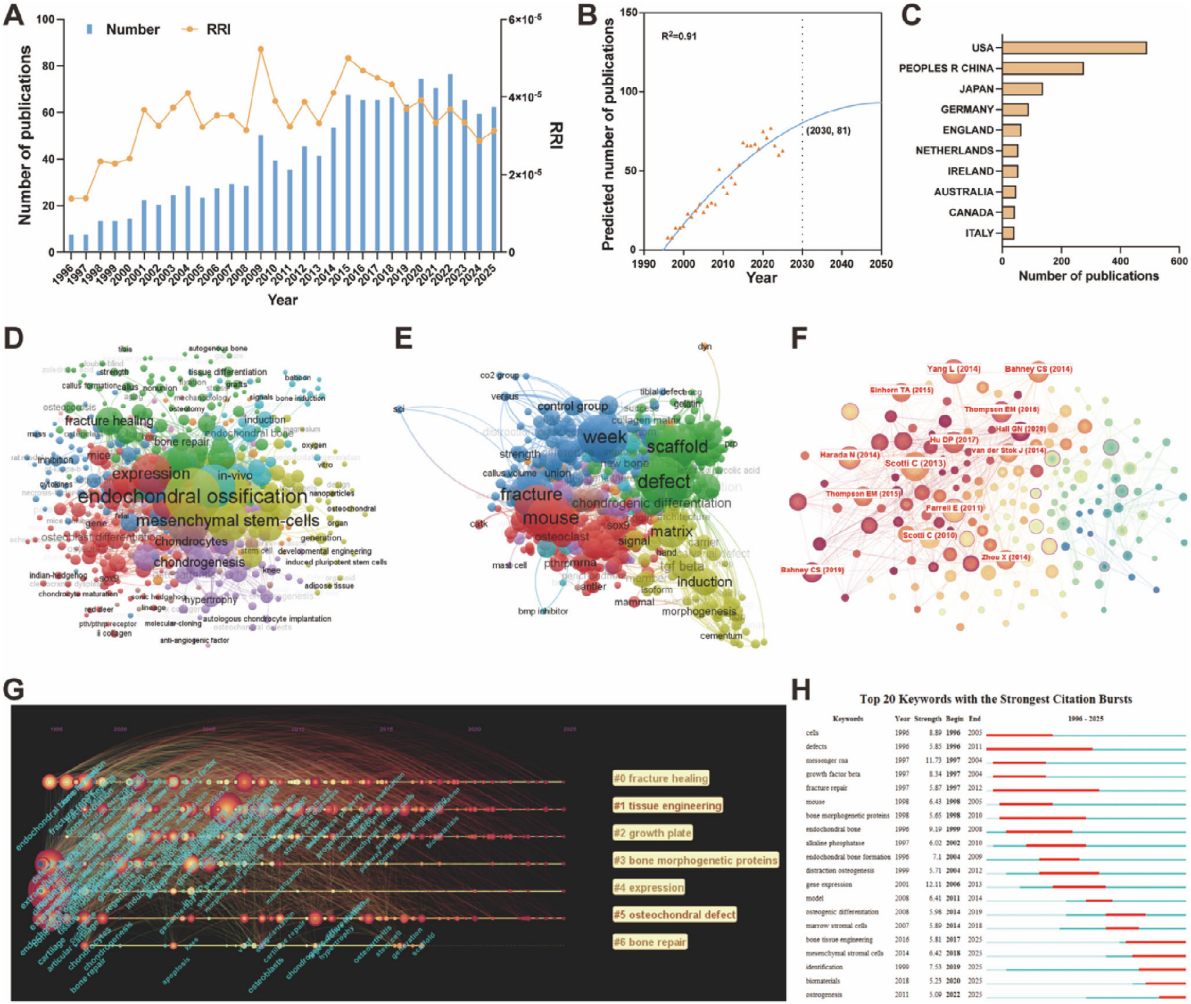
Research trends in the field. (A) Annual number of publications and RRI. (B) Model fitting curves of global trends in publications. (C) Number of publications in the top 10 countries/regions. Analyses in panels (A–C) were performed using GraphPad Prism. (D) Co‐occurrence analysis of keywords by VOSviewer. (E) Co‐occurrence analysis of terms in the main texts (excluding titles and abstracts) by VOSviewer. (F) Co‐citation analysis of references by CiteSpace. (G) Timeline map of keywords from 1996 to 2025 by CiteSpace. (H) Top 20 keywords with the strongest Citation Bursts by CiteSpace.

According to the retrieval strategy, we obtained a total of 1280 publications that meet the criteria. The publication volume on ECO in bone repair has gradually increased from less than 10 publications in 1996 to over 60 publications in recent years, with the highest number of studies published in 2022 (77 publications). The relative research interest (RRI), defined as the ratio of the number of publications in a specific field to the total number of publications across all fields in a given year [321], exhibited year‐to‐year fluctuations but an overall upward trend, indicating a growing research interest in this field (Figure 7A). To predict the future trends in global literature publication, we created a time curve of publication volume using a logistic regression model based on the publication situation over the past thirty years (R‐square = 0.91). According to this time curve, we predict that the publication volume in 2030 will reach 81 papers (Figure 7B). The United States has made the largest contribution in this field, followed by China, Japan, and Germany (Figure 7C).

We further visualize the keywords of recent publications. In the co‐occurrence analysis of keywords, besides “endochondral ossification,” frequently occurring keywords include “bone,” “cartilage,” “differentiation,” “mesenchymal stem cells,” “chondrogenesis,” “expression,” and “angiogenesis,” all closely related to the biological process of ECO (Figure 7D). In addition, we conducted a co‐occurrence analysis of terms within the text, which identified “mouse,” “fracture,” “scaffold,” “defect,” and “week” as core high‐frequency terms(Figure 7E). These findings indicate that research in this field predominantly relies on animal models, particularly mouse models, and focuses on representative disease models such as bone defects and fracture repair. Moreover, scaffold‐based strategies are widely employed to evaluate bone regeneration processes and repair outcomes, reflecting a relatively mature and well‐established research paradigm in this field. We further performed a co‐citation analysis of the literature (Figure 7F). Among publications over the past three decades, the most highly cited article was “Engineering of a functional bone organ through endochondral ossification” by Scotti et al., published in *Proc Natl Acad Sci USA* in 2013 [20]. This study first reported a novel engineered large‐scale human ECO organoid generated in vitro, providing an important experimental foundation and methodological framework for bone regeneration research based on ECO strategies.

We further constructed a timeline view of these keywords (Figure 7G), which were grouped into six major clusters: fracture healing, tissue engineering, growth plate, bone morphogenetic proteins, expression, osteochondral defect, and bone repair. The timeline clearly illustrates the temporal evolution of research hotspots within each cluster. For example, studies on gene expression and BMP‐related mechanisms were primarily concentrated in the early period, whereas the focus on tissue engineering and osteochondral defect repair has increased since the turn of the century. Meanwhile, growth plate and fracture healing have maintained long‐term, stable attention. Additionally, we listed the top 20 keywords with the highest citation burst strength (Figure 7H). Keywords that exhibited early citation bursts were mainly related to fundamental mechanistic studies, whereas more recent bursts, such as “bone tissue engineering,” “mesenchymal stromal cells,” and “biomaterials,” reflect increasing attention to translational and engineering applications, further highlighting the shift from mechanistic research toward tissue engineering applications.

## 7 Conclusion and Perspectives

This review systematically summarizes the research progress on ECO, covering not only the biological processes and signaling regulatory mechanisms of ECO, but also the types of seed cells, biomaterial scaffolds, key bioactive factors, and strategies for in vitro organoid construction. It further proposes future optimization directions based on current limitations. In addition, through bibliometric analysis, the review quantitatively presents research hotspots and development trends, providing researchers with a clear knowledge framework and roadmap for future studies.

Based on a systematic overview from fundamental mechanisms to engineering translation, future developments can be advanced in several aspects. On one hand, it is necessary to further optimize organoid construction strategies to enhance the efficiency and temporal sequence of cartilage formation and osteogenic differentiation, while better coordinating multiple cell types and precisely regulating the 3D microenvironment. On the other hand, the regulatory roles of signaling pathways and the microenvironment on ECO should be further elucidated to improve the functionality and stability of engineered tissues. In addition, combining smart scaffold materials with bioactive factors to achieve precise spatiotemporal control of cell behavior will provide new strategies for constructing more mature organoid systems. Finally, promoting the translation of ECO from in vitro models to in vivo validation and clinical applications is also crucial, including applications in bone defect repair, intervention of degenerative joint diseases, and the development of drug screening platforms. Overall, future ECO research is expected to progress toward more controllable, functional, and clinically translatable directions.

Looking ahead, ECO research presents multiple exciting avenues for development. The construction of ECO organoids and organ‐on‐chip systems in vitro provides not only feasible models for bone defect repair but also new platforms for drug screening and disease mechanism studies. The combination of novel biomaterials and smart scaffolds is expected to enhance the structural maturation and functionality of engineered tissues, while gene editing and signaling modulation technologies may enable the creation of customized ECO models. Overall, the continuous optimization of ECO engineering strategies not only drives innovation in bone regeneration approaches but also expands the potential applications across other medical fields.

## Author Contributions

Y.Q.S. and Z.H.H. contributed equally to this work and were responsible for conceptualization, investigation, methodology, formal analysis, visualization, and writing – original draft data. Q.Q.C. and L.C. contributed to the investigation, formal analysis, visualization, and writing – original draft data. C.Y.W. and Z.J.Z. were involved in methodology, software, and data curation. J.H.L. was responsible for project administration, conceptualization, funding acquisition, and manuscript revision. H.L. and D.X., as corresponding authors, were responsible for project administration, conceptualization, funding acquisition, resources, validation and manuscript revision. All authors have read and approved the final manuscript.

## Funding

This research was funded by the National Key Research and Development Program of China (2024YFA1108603), Natural Science Foundation of Beijing Municipality (L222087, L232094, and L244019), National Natural Science Foundation of China (82302776), and Peking University People's Hospital Scientific Research Development Funds (RS2024‐04).

## Conflicts of Interest

The authors declare no conflicts of interest.

## Data Availability

No new datasets were generated during the current study. Data used for the bibliometric analysis were retrieved from the Web of Science Core Collection (WOSCC).
